# *In vivo* imaging reveals mitophagy independence in the maintenance of axonal mitochondria during normal aging

**DOI:** 10.1111/acel.12654

**Published:** 2017-08-07

**Authors:** Xu Cao, Haiqiong Wang, Zhao Wang, Qingyao Wang, Shuang Zhang, Yuanping Deng, Yanshan Fang

**Affiliations:** ^1^ Interdisciplinary Research Center on Biology and Chemistry Shanghai Institute of Organic Chemistry Chinese Academy of Sciences Shanghai China; ^2^ University of Chinese Academy of Sciences Shanghai China

**Keywords:** aging, axonal integrity, fission–fusion, *in vivo* imaging, mitochondria, mitophagy

## Abstract

Mitophagy is thought to be a critical mitochondrial quality control mechanism in neurons and has been extensively studied in neurological disorders such as Parkinson's disease. However, little is known about how mitochondria are maintained in the lengthy neuronal axons in the context of physiological aging. Here, we utilized the unique *Drosophila* wing nerve model and *in vivo* imaging to rigorously profile changes in axonal mitochondria during aging. We revealed that mitochondria became fragmented and accumulated in aged axons. However, lack of *Pink1* or *Parkin* did not lead to the accumulation of axonal mitochondria or axonal degeneration. Further, unlike in *in vitro* cultured neurons, we found that mitophagy rarely occurred in intact axons *in vivo*, even in aged animals. Furthermore, blocking overall mitophagy by knockdown of the core autophagy genes *Atg12* or *Atg17* had little effect on the turnover of axonal mitochondria or axonal integrity, suggesting that mitophagy is not required for axonal maintenance; this is regardless of whether the mitophagy is PINK1‐Parkin dependent or independent. In contrast, downregulation of mitochondrial fission–fusion genes caused age‐dependent axonal degeneration. Moreover, *Opa1* expression in the fly head was significantly decreased with age, which may underlie the accumulation of fragmented mitochondria in aged axons. Finally, we showed that adult‐onset, neuronal downregulation of the fission–fusion, but not mitophagy genes, dramatically accelerated features of aging. We propose that axonal mitochondria are maintained independently of mitophagy and that mitophagy‐independent mechanisms such as fission–fusion may be central to the maintenance of axonal mitochondria and neural integrity during normal aging.

## Introduction

Healthy mitochondria are critical for maintaining normal bioenergetically demanding activities of neurons. Such energy demand in neuronal axons is likely to be especially extreme due to the activities such as synaptic transmission, generating and propagating action potentials, and transporting biomaterials over a long distance. Deleterious mitochondrial changes such as a decrease in mitochondrial integrity and function are associated with aging and neurodegenerative diseases (Bratic & Larsson, 2013; López‐Otín *et al*., 2013). Mitophagy is an important mitochondrial quality control mechanism that selectively eliminates damaged mitochondria by autophagy (Wang and Klionsky, 2014; Wei *et al*., 2015). The process is regulated by the PTEN‐induced putative kinase 1 (PINK1) and the E3 ubiquitin ligase Parkin (Pickrell & Youle, 2015), whose mutations can cause familial forms of Parkinson's disease (PD).

It is widely assumed that damaged mitochondria accumulate in neurons if mitophagy fails, which causes PD in PINK1‐Parkin deficiency. For axons, the studies based on *in vitro* cultured neurons have been controversial, especially regarding whether mitophagy occurs locally in axons. PINK1‐Parkin‐mediated mitophagy was reported to occur in neurons but restricted to the somatodendritic regions (Seibler *et al*., 2011; Cai *et al*., 2012). However, Maday *et al*. (2012); Maday & Holzbaur (2014) showed that autophagosomes were generated at neurite tips, which fused with axonal mitochondria and together were transported to the neuronal soma for lysosome‐mediated degradation. By contrast, another study reported that autophagosomes were readily available in axons and were recruited to damaged mitochondria in a PINK1‐Parkin‐dependent manner followed by local turnover in axons (Ashrafi *et al*., 2014). Other than by inference, evidence *in vivo* supporting an essential role of PINK1‐Parkin in regulating mitochondrial quality control in axons during aging is lacking. As axonal mitochondria may behave differently in neurons cultured *in vitro* (Sung *et al*., 2016), it is important to elucidate how mitochondria are maintained in the lengthy axons under physiological conditions *in vivo*.

Mitochondria are dynamic organelles that undergo constant fission and fusion. The fission–fusion balance is critical for determining mitochondrial morphology, distribution, abundance, and function (Chan, 2012), and allows mitochondria to quickly adapt to different metabolic needs in different subcellular compartments or upon different energetic states (Wai & Langer, 2016). Moreover, fission–fusion is also an important means to control mitochondrial quality, via removing the damaged portion of a mitochondrion by fission or via refreshing a dysfunctional mitochondrion by fusion to a healthy one (Schrepfer & Scorrano, 2016). The key regulators of mitochondrial dynamics are an evolutionarily conserved family of dynamin‐related GTPases (Chan, 2012). Specifically, mitofusin‐1 (Mfn1) and ‐2 (Mfn2) mediate fusion of the mitochondrial outer membrane and optic atrophy 1 (Opa1) mediates that of the inner membrane; fission is mainly controlled by dynamin‐related protein 1 (Drp1) (Chan, 2012; Bertholet *et al*., 2016). However, the role of mitochondrial fission–fusion, or its relative contribution compared to that of mitophagy, in axonal mitochondrial quality control *in vivo* is unknown.

Dissecting the mechanism maintaining axonal mitochondria in animals during their lifetime has been challenging, which is at least in part due to the limitation of classical sample fixation and staining processes that grossly alter mitochondrial morphology. In this regard, the *Drosophila* wing model that we developed provides a unique system (Fang *et al*., 2012, 2013; Fang & Bonini, 2015) that allows direct visualization of the morphology of axonal mitochondria under near physiological conditions *in vivo*. Using this system, we critically evaluate the role of PINK1‐Parkin‐dependent and PINK1‐Parkin‐independent mitophagy as well as mitochondrial fission–fusion in maintaining axonal integrity during aging. We find that fragmented mitochondria accumulate in aged axons, which is consistent with the general idea that mitochondrial quality and function decline with age (Bratic & Larsson, 2013; López‐Otín *et al*., 2013). However, our study reveals a striking scarcity and dispensability of axonal mitophagy in neuronal aging, whether PINK1‐Parkin dependent or independent. Instead, our findings raise the possibility that mitophagy‐independent mechanisms such as fission–fusion play a central role in the maintenance of axonal mitochondria during normal aging.

## Results

### *In vivo* neuroimaging reveals morphological alterations of axonal mitochondria during aging

**Figure 1:**
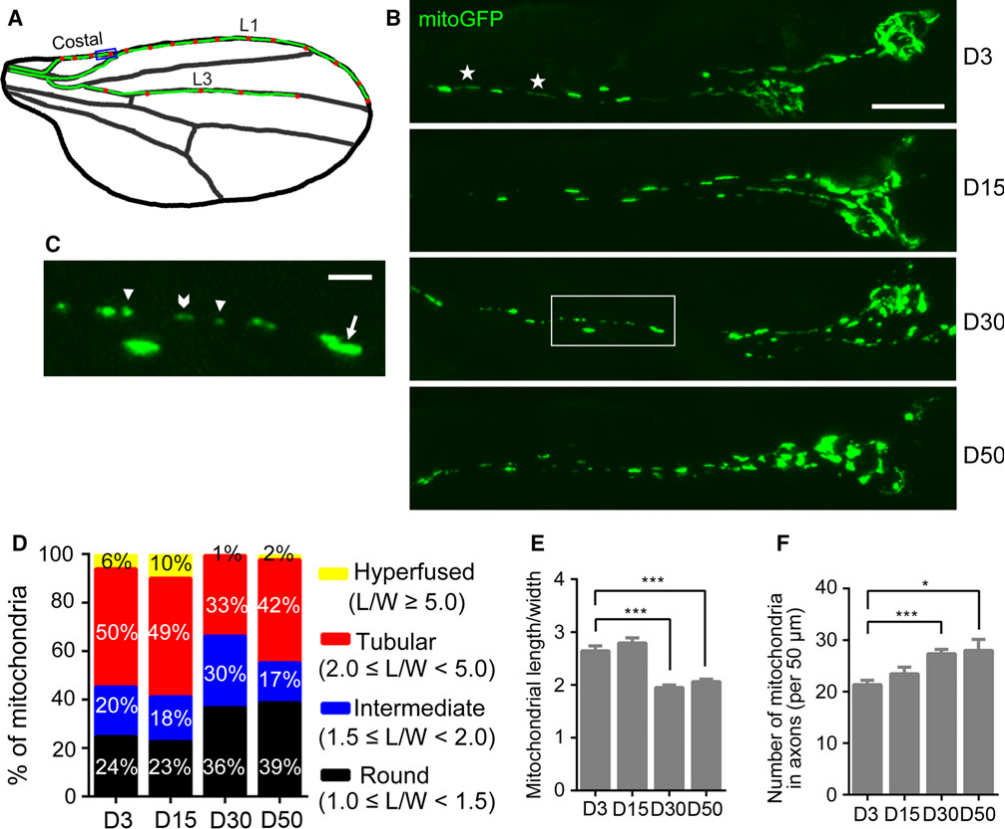
*In vivo* neuroimaging reveals the accumulation of fragmented mitochondria in aged axons. (A) A cartoon illustration of the *Drosophila* wing. The green line highlights the wing nerve in the costal, L1, and L3 wing veins, and the red dots denote the neuronal soma. (B, C) Representative confocal images of mitochondria in the axons of the *Drosophila* costal wing nerve (the blue box in A) at days D3, D15, D30, and D50. Mitochondria are labeled by mitoGFP using a *dpr*‐Gal4 driver. (C) A higher magnification of the area in the white box in B. Arrowheads: short; chevron: intermediate; arrow: tubular; and asterisks: hyperfused axonal mitochondria. (D) The axonal mitochondria imaged in (B) are classified into four groups based on the length/width ratio as specified. (E, F) The average length/width ratio (E) and number (F) of axonal mitochondria at indicated ages. Data are shown as mean ± SEM,* n* = 9–16 wings per group. *P <* *0.05, ***P <* *0.001. Scale bars, 10 μm in (B) and 2 μm in (C).

To characterize the dynamic morphological changes in neuronal mitochondria during aging, we used the GAL4/UAS system to label neuronal mitochondria with mitochondria‐localized GFP (mitoGFP) in the *Drosophila* wing nerve. The flies were aged to different time points and the axons were imaged in *live* fly wings without fixation by confocal microscopy. Mitochondria in the neuronal soma and axons of the costal wing nerve (the boxed area in Fig. 1A) were directly visualized *in vivo* (Fig. 1B). The axonal mitochondria displayed a mixed population of morphology, from round or almost round (1.0 ≤ length/width < 1.5), intermediate (1.5 ≤ length/width < 2.0), tubular (2.0 ≤ length/width < 5.0), to hyperfused (length/width ≥ 5) (Fig. 1C,D). We quantified the length and width of each mitochondrion in the costal wing nerve, and calculated the average length/width ratio at different ages in Fig. 1E. It is evident that both the average length/width ratio and the proportion of long mitochondria (tubular and hyperfused) were decreased, whereas those of short mitochondria (intermediate and round) were increased in aged flies (D30 and D50). Concurrently, the number of axonal mitochondria was increased with age (Fig. 1F). These data suggest that mitochondria became fragmented and accumulated in aged axons.

### The PINK1‐Parkin pathway is dispensable for axonal maintenance in both sensory and motor neurons in adult *Drosophila*

**Figure 2:**
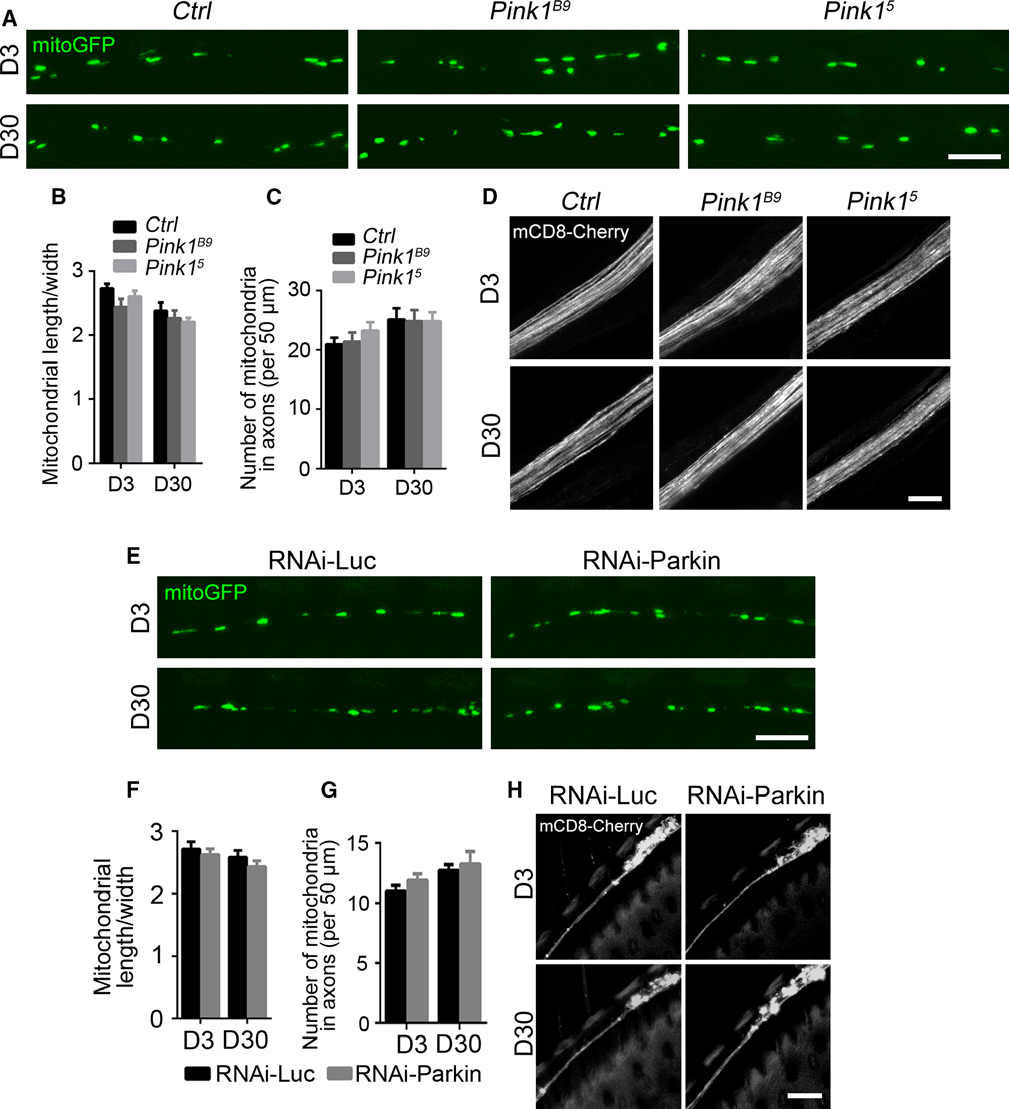
Flies with Pink1‐Parkin deficiency exhibit normal mitochondrial morphology and intact axonal integrity. (A) Axonal mitochondria labeled by mitoGFP in the costal wing nerve of control (*w* ^*1118*^), *Pink1* ^*B9*^ *,* and *Pink1* ^*5*^ flies at D3 or D30. (B‐C) Quantification of length/width ratio (B) and the number (C) of the axonal mitochondria. (D) Representative *in vivo* images of distal axons of the L1 wing nerve of control (*w* ^*1118*^), *Pink1* ^*B9*^ *,* and *Pink1* ^*5*^ flies at D3 or D30. (E) Representative images of axonal mitochondria of RNAi‐*Luc* (control) and RNAi‐*Parkin* flies at D3 or D30, the average length/width ratio, and the number of axonal mitochondria are quantified in (F, G). Data are shown as mean ± SEM,* n* = 8–10 wings per group. Two‐way ANOVA shows no statistically significant difference between any of the mutant or RNAi groups and their respective controls, whereas the mitochondrial changes with age are significant (*P *<* *0.05) except for (F). (H) Representative *in vivo* images of neuronal soma and proximal axons of the L1 nerve of indicated genotypes at D3 and D30. No axonal degeneration is detected. Scale bars, 5 μm in (A) and (E), and 10 μm in (D) and (H).

The accumulation of fragmented mitochondria in axons might be a result of declined mitochondrial turnover during aging. Damaged axonal mitochondria in cultured neurons could be cleared by local mitophagy, which requires the function of PINK1 and Parkin (Ashrafi *et al*., 2014). Hence, we first investigated the contribution of the PINK1‐Parkin pathway to the age‐associated accumulation of axonal mitochondria *in vivo*. The *Pink1* loss‐of‐function (LOF)‐mutant flies, *Pink1* ^*B9*^ and *Pink1* ^*5*^, exhibited collapsed thorax and abnormal wing posture (Fig. S1A,B, Supporting information), which was consistent with the mitochondrial defects in muscles as previously reported (Clark *et al*., 2006; Park *et al*., 2006). Unlike in muscles, the morphology of axonal mitochondria in the wing axons of *Pink1* ^*B9*^ and *Pink1* ^*5*^ flies looked mostly normal (Fig. 2A). Neither the average mitochondrial length/width ratio nor the number of mitochondria showed a significant difference between *Pink1* mutants and *w* ^*1118*^ control flies (Fig. 2B,C). Moreover, the axonal integrity of the *Pink1* mutants was well maintained and no axonal degeneration was observed during aging (Fig. 2D).

Similarly, despite an age‐associated decrease in *Parkin* expression (Fig. S1C, Supporting information), downregulation of *Parkin* in the wing nerve did not result in the accumulation of fragmented mitochondria (Fig. 2E–G) or axonal degeneration (Fig. 2H). The knockdown (KD) efficiency was examined by quantitative RT–PCR (qPCR) (Fig. S1D, Supporting information). In contrast, downregulation of *Parkin* or *Pink1* in muscles using the same transgenic RNAi strains caused muscle degeneration and collapsed thoraces (Fig. S1E, Supporting information), confirming their essential role in the muscle cells. As the fly wing nerve consists of only sensory neurons (Nakamura *et al*., 2002), we asked whether the lack of axonal degeneration in PINK1‐Parkin deficiency was sensory neuron‐specific. We then examined the axons of the motor neurons in the adult *Drosophila* leg (Fig. S2A, Supporting information). Similar to the sensory wing nerve, the motor neurons of the *Pink1* LOF mutants showed no axonal degeneration, even in aged flies (Fig. S2B, Supporting information).

As overexpression of *Parkin* in adult neurons was shown to enhance mitochondrial activity and extend longevity in *Drosophila* (Rana *et al*., 2013), we were keen to test whether upregulation of PINK‐Parkin could promote turnover of fragmented mitochondria and improve axonal maintenance during aging. Unexpectedly, overexpression of *Pink1* or *Parkin* in the wing neurons led to extremely fragmented mitochondria (Fig. S3A,B, Supporting information). Expression of the enzymatically inactive mutants, *Pink1* ^*L464P*^ (Song *et al*., 2013), *Parkin* ^*T240R*^ *,* or *Parkin* ^*R275W*^ (Lee *et al*., 2010; Kim *et al*., 2013) did not show such effect, indicating that the impact of upregulating PINK1‐Parkin on axonal mitochondrial morphology requires their enzymatic activity. Furthermore, in striking contrast to LOF and KD of *Pink1* and *Parkin* (Fig. 2D,H), the wing nerve of *Pink1‐* or *Parkin*‐overexpressing flies degenerated rapidly with age (Fig. S3C,E, Supporting information). This is likely due to a mitophagy‐independent function of Parkin in regulating Marf, the *Drosophila* homologue of mammalian mitofusins that promote mitochondria fusion (Chan, 2012; Schrepfer & Scorrano, 2016; Fig. S3A, Supporting information). Parkin was known to ubiquitinate Marf for proteasome‐mediated degradation and resulted in shortened mitochondria in insect cells (Poole *et al*., 2010; Ziviani *et al*., 2010). Consistently, we found that both mitochondrial morphology (Fig. S3A,B, Supporting information) and axonal integrity (Fig. S3D,E, Supporting information) of *Parkin*‐overexpressing neurons were significantly improved by co‐expression of *Marf*. This result is in line with the idea that mitophagy‐independent PINK1‐Parkin functions may cause or contribute to the pathogenesis of PD (Pryde *et al*., 2016).

### Mitophagy rarely occurs in intact axons *in vivo* during normal aging

**Figure 3:**
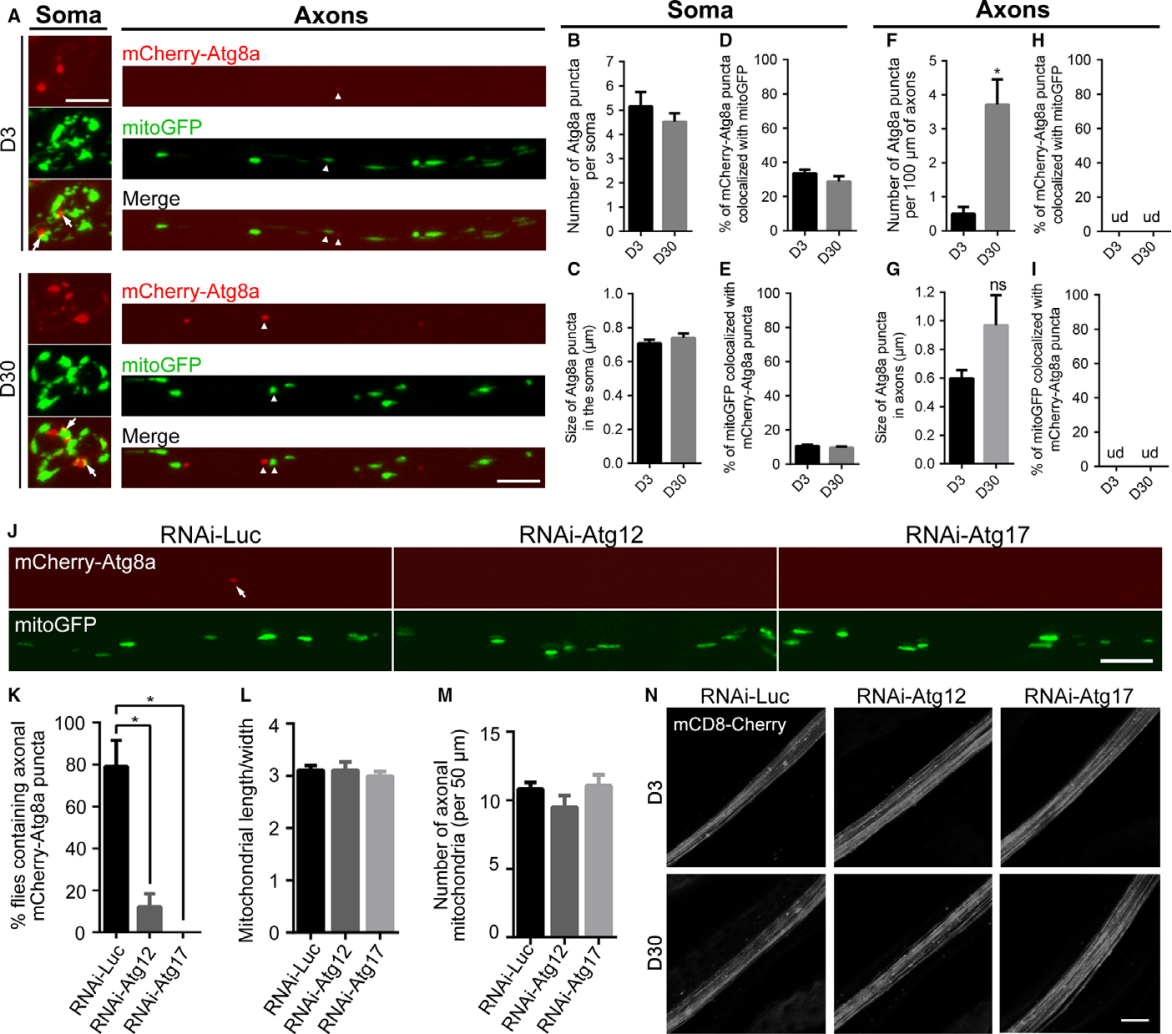
Mitophagy is both rare and dispensable for intact axons *in vivo* during aging. (A–I) Axonal mitophagy rarely occurs *in vivo* under physiological conditions. (A) Representative images showing neuronal soma (left) and axons (right) with autophagosomes (AP) labeled by mCherry‐Atg8a and mitochondria labeled by mitoGFP. mCherry‐Atg8a puncta are commonly seen in the soma of both young and aged flies; axonal AP are rarely observed in young flies but significantly increase with age. Arrows, colocalization of mitoGFP and mCherry‐Atg8a puncta in the soma; arrowheads, nearby but not colocalized mitochondria and autophagosome in axons. The number and size of somatic AP (B‐C) and axonal AP (F‐G) as well as colocalization of mitoGFP and mCherry‐Atg8a in the soma (D, E) are quantified. Colocalization of mitochondria and autophagosomes is almost never detected in axons *in vivo* even in aged animals; hence, the colocalization occurrence is essentially zero in (H, I). Data are shown as mean ± SEM,* n* = 7–12 wings per group. *P <* *0.05; ns, not significant; ud, undetected. (J–N) Blocking axonal autophagy has little effect on the maintenance of axonal mitochondria or axonal integrity *in vivo*. (J) Representative images showing AP (mCherry‐Atg8a, arrow) and mitochondria (mitoGFP) in the costal wing axons of RNAi‐*Luc*, RNAi‐*Atg12*, and RNAi‐*Atg17* flies. (K) The percentage of flies containing axonal AP in the entire wing nerve. (L, M) Quantification of the length/width ratio (L) and the number (M) of the axonal mitochondria. Data are shown as mean ± SEM,* n* = 11–14 wings per group. *P <* *0.05. (N) Representative images of the distal wing nerve of the indicated genotypes at D3 and D30. No sign of axonal degeneration is observed in the RNAi‐*Atg12* or RNAi‐*Atg17* flies. Scale bars, 5 μm in (A) and (J), and 10 μm in (N).

In addition to the PINK1‐Parkin‐dependent mitophagy, mitophagy can take place independently of PINK1‐Parkin (Wei *et al*., 2015). Hence, the accumulation of axonal mitochondria might also be due to an age‐associated decline of autophagic clearance of damaged mitochondria that is independent of PINK‐Parkin. To test this possibility, we generated the fly strains to express mCherry‐labeled Atg8a (an homologue of mammalian LC3) in the wing nerve to visualize and investigate autophagy by *in vivo* imaging. Induction of autophagy leads to the cleavage and translocation of Atg8a to the autophagosome membrane, which transforms mCherry‐Atg8a signal from diffuse to punctate. Formation of Atg8a/LC3 puncta is widely used as an autophagosome marker (Klionsky *et al*., 2016). As illustrated in Fig. 3A, in the neuronal soma, autophagosomes (mCherry‐Atg8a puncta) were frequently observed in both young (D3) and aged (D30) flies. The neuronal soma maintained relatively constant basal levels of autophagy during aging, as neither the number nor the size of mCherry‐Atg8a puncta was significantly changed in the soma during aging (Fig. 3B,C). In contrast, autophagosome puncta were sparse in young axons. With age, an increasing number of mCherry‐Atg8a puncta were detected in axons (Fig. 3A,F); the size of axonal autophagosomes showed a trend to increase although this was not statistically significant (Fig. 3G). These results suggested an increase in autophagy induction and/or a decrease in autophagic flux in axons during aging.

As the numbers of fragmented mitochondria and autophagosomes were both increased in aged axons, we asked whether this concurrence represented an increase in mitophagy in axons. A previous study showed that depolarized axonal mitochondria became colocalized with autophagosomes, leading to local mitophagy in axons (Ashrafi *et al*., 2014). Hence, we carefully examined colocalization of mitoGFP and mCherry‐Atg8a puncta in the wing nerve. Unexpectedly, however, we found mitoGFP and mCherry‐Atg8a puncta were essentially not colocalized *in vivo*, even in aged flies (Fig. 3A, arrowheads). Quantification showed that neither the proportion of mCherry‐Atg8a puncta containing mitochondria nor the percentage of mitochondria colocalized with mCherry‐Atg8a changed significantly during aging (Fig. 3H–I). Of note, among over 110 fly wings examined in this study, we only detected two individual cases of a mitochondrion colocalized with mCherry‐Atg8a puncta in the entire wing nerve, which is composed of more than 200 axons (Fang *et al*., 2013). As such, the occurrence of axonal mitophagy under physiological conditions *in vivo* is <1 of 10 000 axons, suggesting that in axons virtually no mitophagy occurs, regardless of age. Similarly, McWilliams *et al*. (2016) reported that the majority of mitochondrial turnover occurred in the soma of Purkinje neurons with minimal mitophagy observed in mouse axons *in vivo*.

### Blocking autophagy in the *Drosophila* wing nerve has a minimal effect on mitochondrial turnover or axonal integrity during aging

Although axonal mitophagy rarely occurred *in vivo*, it did not exclude the possibility that occasional axonal mitophagy played an essential role. As both PINK1‐Parkin‐dependent and PINK1‐Parkin‐independent mitophagy pathways rely on the autophagy machinery for the final clearance of mitochondria, we sought to examine the effect of blocking autophagy on mitochondrial turnover and axonal integrity during aging.

Autophagy is controlled by a family of evolutionarily conserved ATG genes. One of the core ATG genes is *Atg12*, required for the formation of autophagosomes in promoting the conjugation of ATG8 protein to the lipid phosphatidylethanolamine (Ktistakis & Tooze, 2016). As the aging context limited the use of the null mutants in this study, we could only assess the impact of these genes in a partial compromise situation. Nevertheless, in the RNAi‐*Atg12* flies, both the number and size of mCherry‐Atg8a puncta in the soma were significantly decreased; meanwhile, diffused mCherry‐Atg8a signal was increased (Fig. S4A–C, Supporting information). The KD efficiency of RNAi‐*Atg12* and RNAi‐*Atg17* was examined in Fig. S4D (Supporting information). Importantly, despite the low basal level of autophagy in axons (Fig. 3J, control flies, RNAi‐*luc*), *Atg12* KD caused a further decrease in axonal autophagy (Fig. 3J, RNAi‐*Atg12*), which was seen as a drastic reduction in flies with detectable mCherry‐Atg8a puncta in axons (Fig. 3K, from ~80% in the control to only ~10% in the RNAi‐*Atg12*). As such, axonal autophagy was inhibited in the wing nerve in the majority of RNAi‐*Atg12* flies. Nonetheless, the maintenance of axonal mitochondria was not impaired, as RNAi‐*Atg12* flies showed no difference in either the average length/width ratio or the density of axonal mitochondria (Fig. 3J,L,M).

The lack of a deleterious effect on axonal mitochondrial turnover was further confirmed by manipulation of another core ATG gene *Atg17* (required for the autophagosome assembly, Ktistakis & Tooze, 2016) in the wing nerve (Fig. 3J–M). In nearly 100% of RNAi‐*Atg17* flies, an axonal autophagosome was never found in over two hundred axons examined in the entire wing nerve (Fig. 3K). Nevertheless, no degeneration of a single axon was observed, even in aged RNAi‐*Atg17* flies (Fig. 3N, D30). These results further support the conclusion that axonal mitochondria do not rely on mitophagy for their maintenance during normal aging.

### Mitophagy‐independent mechanisms may regulate mitochondrial maintenance and axonal integrity during aging

**Figure 4:**
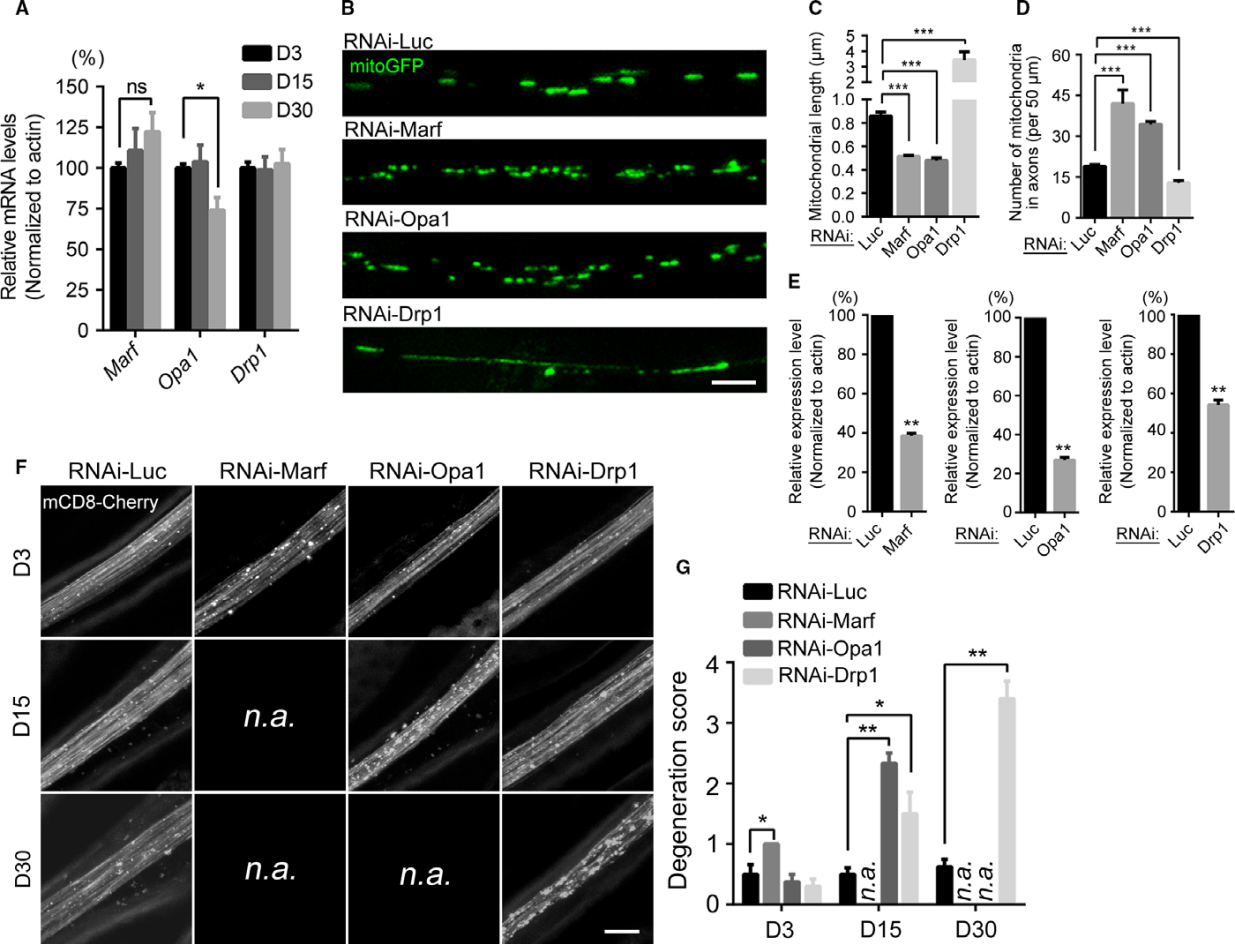
The fission–fusion balance is critically required for the maintenance of axonal mitochondria and axonal integrity during aging. (A) The relative mRNA levels of mitochondrial fission–fusion genes in the fly head at the indicated time points. *Opa1* expression is significantly decreased at D30. (B–D) Representative images of axonal mitochondria of indicated genotypes, and the axonal mitochondrial length and number are quantified in (C) and (D), respectively. Data are shown as mean ± SEM,* n* = 8–14 wings per group except for RNAi‐*Marf*, of which only three adult flies were available for imaging due to early lethality. (E) The knockdown efficiency of the above RNAi lines was examined by qPCR. (F, G) Age‐dependent axonal degeneration by downregulation of the fission–fusion genes in the adult fly wing nerve. The representative images of the L1 wing nerve of indicated genotypes at D3, D5, and D30 are shown in (F) and the degeneration scores are quantified in (G) as described in the Methods. Data are shown as mean ± SEM,* n* = 6–8 wings per genotype per time point except for RNAi‐*Marf* (*n* = 3). *n.a*., not available; because KD of *Marf* or *Opa1* by the *dpr*‐Gal4 driver (which is highly expressed in the adult wing neurons that facilitates the *in vivo* imaging but is also expressed in neurons elsewhere) causes early lethality in young adults, the imaging data at later time points for RNAi‐*Marf* and RNAi‐*Opa1* flies are unavailable. *P <* *0.05, **P <* *0.01, ***P <* *0.001; ns, not significant. Scale bars, 2 μm in (B) and 10 μm in (F).

Neuronal integrity and functions rely on mitochondria for ATP production. Because we revealed that mitophagy was dispensable for axons under physiological conditions *in vivo*, neurons must use other means to maintain axonal mitochondria and axonal integrity. Mitochondrial quality and abundance is also controlled by mitochondrial transport and fission–fusion (Saxton & Hollenbeck, 2012; Schrepfer & Scorrano, 2016). Indeed, we and others previously showed that proper mitochondrial transport and distribution are essential for the integrity of *Drosophila* axons (Avery *et al*., 2012; Fang *et al*., 2012). As fission–fusion also regulates the morphology and turnover of mitochondria (Chan, 2012; Schrepfer & Scorrano, 2016), we next investigated the possibility that the accumulation of fragmented mitochondria in aged axons resulted from an age‐associated change in the fission–fusion dynamics. Interestingly, we found that *Opa1* expression in the fly head became reduced with age (Fig. 4A, D30). However, as a similar expression decrease was detected with *Parkin* (Fig. S1C, Supporting information), but KD of *Parkin* did not impair mitochondrial turnover or axonal integrity (Fig. 2E–H), it was yet to be proven that fission–fusion was indeed essential for axonal maintenance during aging.

As shown in Fig. 4B–D, downregulation of *Marf* or *Opa1* significantly decreased, whereas KD of *Drp1* increased, the length of axonal mitochondria in the wing nerve. We then examined the impact on axonal integrity. RNAi‐*Marf* flies showed remarkable axonal degeneration as early as D3, RNAi‐*Opa1* flies at D15, and RNAi‐*Drp1* flies at D30 (Fig. 4E). Of note, because KD of *Marf* or *Opa1* using the *dpr*‐Gal4 driver (which is highly expressed in the adult wing neurons to facilitate *in vivo* imaging, but is also expressed in neurons elsewhere) caused early lethality of young adults, the axonal imaging data at later time points for RNAi‐*Marf* (D15 and D30) and RNAi‐*Opa1* flies (D30) were unavailable. Nevertheless, downregulation of the fission–fusion genes by genetic manipulations caused age‐dependent, progressive axonal degeneration. This result suggests that the change in their expression during normal aging should not be overtly large; otherwise, aging itself would cause spontaneous axonal degeneration. Indeed, the small (~30%) but significant reduction in *Opa1* expression in aged neurons (Fig. 4A) is consistent with the moderate alteration of mitochondrial morphology in axons during normal aging.

### Inhibition of fission–fusion but not mitophagy in adult neurons significantly accelerates aging in *Drosophila*

**Figure 5:**
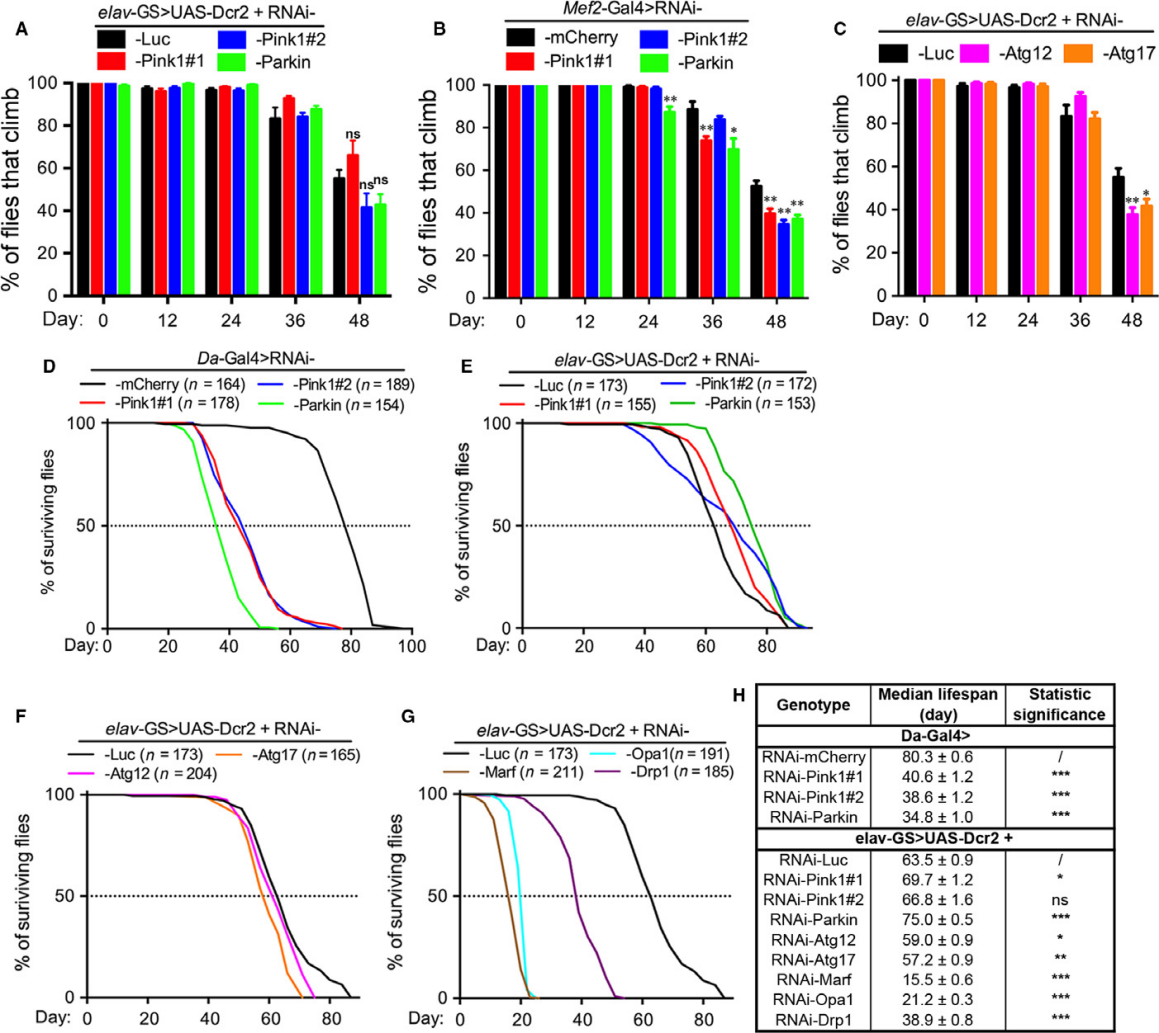
Inhibition of fission–fusion but not mitophagy in adult neurons significantly accelerates aging. (A‐C) Climbing assays of flies with (A) *Pink1* or *Parkin* downregulated in adult neurons by *elav‐* GS, (B) in muscles by *Mef2*‐Gal4 (B), or (C) *Atg12* or *Atg17* downregulated in adult neurons. The locomotive ability was assessed as the average percentage of flies climbing over 5 cm within 15 s. Data are shown as mean ± SEM,* n* = 20 flies per vial and 6–8 vials each group. *P <* *0.05, **P <* *0.01; ns, not significant. (D) Knockdown of *Pink1* or *Parkin* in all cells by the *Da*‐Gal4 driver dramatically reduces the longevity. (E‐G) Lifespan assays of the flies with adult‐onset, neuronal downregulation (*elav*‐GS) of *Pink1* or *Parkin* (E), *Atg12* or *Atg17* (F), or *Marf*,* Opa1,* or *Drp1* (G). A copy of UAS‐*Dcr2* was co‐expressed to boost the RNAi knockdown efficiency in neurons (Ni *et al*., 2008), but was not needed with the *Mef2*‐Gal4 or *Da*‐Gal4 drivers, in which cases the RNAi‐*mCherry* was used as a control. *n*, the number of flies tested for each genotype is indicated. (H) Summary of the median lifespans, shown as mean ± SEM, *P <* *0.01, **P <* *0.001, ***P <* *0.0001.

It was possible that disruption of the PINK1‐Parkin pathway or the autophagy machinery led to functional deficits before morphological changes were detectable. The climbing assay is widely used to evaluate the function of neurons and muscles in *Drosophila* models of neurodegenerative diseases including PD. As the function of mitophagy/autophagy in neural development might compromise the analysis, we used an inducible, pan‐neuronal driver *elav*‐GeneSwitch (*elav*‐GS, Osterwalder *et al*., 2001) and induced the expression of the RNAi transgenes at adulthood only. As shown in Fig. 5A, no significant difference in the climbing ability was found in RNAi‐*Pink1* or RNAi‐*Parkin* flies. In contrast, KD of *Pink1* or *Parkin* in muscles (*Mef2*‐Gal4) caused age‐dependent climbing defects as early as D24 in RNAi‐*Parkin* and D36 in RNAi‐*Pink1* flies (Fig. 5B). In addition, KD of the core ATG gene *Atg12* or *Atg17* in adult neurons also led to accelerated decline of climbing ability (Fig. 5C). This was even more dramatic when the flies were raised at an elevated environmental temperature of 29 °C (Fig. S4E, Supporting information). Heat stress is known to disturb the proteostasis and require the function of autophagy to maintain the homeostasis (Dokladny *et al*., 2015). Thus, although the autophagy machinery is dispensable for the turnover of axonal mitochondria (Fig. 3J), these results confirmed the essential role of autophagy in neuronal aging likely due to the important functions in regulating the proteostasis in neurons.

**Figure 6:**
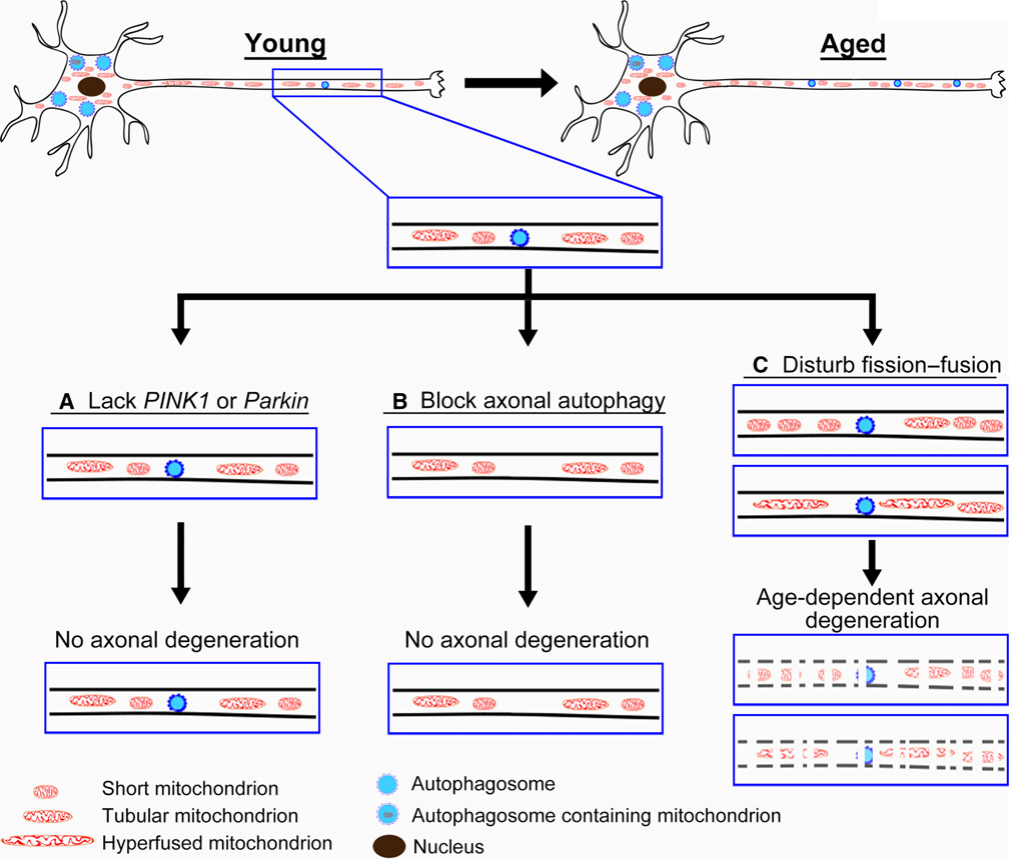
A schematic model of differential requirements of mitophagy and fission–fusion in the maintenance of axonal mitochondria and axonal integrity during aging. During aging, fragmented mitochondria accumulate in axons and the basal levels of axonal autophagy increase. However, mitophagy (evident by the colocalization of mitochondria and autophagosomes) is predominantly observed in the soma but rarely in axons *in vivo*, even in aged neurons. Further, disrupting the PINK1‐Parkin pathway (A) or blocking axonal autophagy by knockdown of the core autophagy genes *Atg12* or *Atg17* (B) does not impair mitochondrial turnover or axonal integrity. Instead, knockdown of the fission–fusion genes *Opa1 Marf* or *Drp1* (C) causes profound changes to the morphology and abundance of axonal mitochondria, which leads to age‐dependent, progressive axonal degeneration and significantly shortens lifespan. Together, our study indicates that mitophagy is dispensable for axonal maintenance *in vivo*, whereas mitophagy‐independent mechanisms such as mitochondrial fission–fusion may play an essential and central role in the maintenance of axonal mitochondria and axonal integrity during normal aging.

Finally, as an attempt to compare the overall involvement of mitophagy and fission–fusion in neuronal aging, we examined the effect of their downregulation on longevity. Similar to their LOF mutants (Greene *et al*., 2003; Cha *et al*., 2005; Clark *et al*., 2006; Park *et al*., 2006), ubiquitous KD of *Pink1* or *Parkin* in all cells using a *Daughterless*‐Gal4 (*Da*‐Gal4) driver dramatically shortened lifespan (Fig. 5D,H). To our great surprise, however, the adult‐onset, neuronal downregulation of *Pink1* or *Parkin* (*elav‐*GS) did not decrease the longevity; instead, the RNAi‐*Parkin* and one of the RNAi‐*Pink1* lines showed a puzzling extension of the lifespan (Fig. 5E,H). In addition, KD of the core autophagy genes *Atg12* or *Atg17* in adult neurons led to a modest reduction in the lifespan (Fig. 5F,H). In contrast, downregulation of the fission–fusion genes in adult neurons all dramatically reduced the longevity (Fig. 5G,H). Of note, disruption of fission–fusion consequently leads to impaired mitochondrial functions (Pich *et al*., 2005), which may add to the deleterious effects on axonal integrity and longevity. Nevertheless, our data demonstrate that mitophagy is dispensable for axonal integrity and physiological aging in neurons, whereas mitophagy‐independent mechanisms such as fission–fusion play an essential role in the maintenance of axonal mitochondria and neural integrity (Fig. 6).

## Discussion

Neuronal aging is known to be associated with deleterious changes in mitochondria including a decrease in mitochondrial biogenesis, the respiratory chain efficacy and ATP generation, an increase in the production of reactive oxygen species, accumulation of mitochondrial DNA mutations, and reduction in mitochondrial transport and turnover (Green *et al*., 2011; López‐Otín *et al*., 2013; Bratic & Larsson, 2013). The development of the *in vivo* imaging paradigm of the *Drosophila* wing nerve has enabled the systematic characterization of mitochondrial morphology in axons during aging in this study. Moreover, taking the advantage of vast genetic tools in *Drosophila*, we have manipulated mitochondrial quality control genes in a spatially and temporally controlled manner, and directly visualized the consequence on mitochondria and axonal integrity *in vivo*. The imaging data show a clear change in mitochondrial heterogeneity from long, tubular mitochondria in young axons toward short, round mitochondria in aged axons. This change is concurrent with an increased number of axonal mitochondria. Thus, aging is associated with an accumulation of fragmented mitochondria in axons. Our finding is consistent with a recent study showing that the axonal mitochondria size decreased in aged *C. elegans* (Morsci *et al*., 2016). This is in addition to the reported decline of axonal mitochondrial transport during aging in nematode neurons (Morsci *et al*., 2016) and mouse retinal ganglion cells (Takihara *et al*., 2015).

Unlike in the neuronal soma where lysosomes are abundant and mitochondria can be readily cleared by mitophagy, mitochondria in distal axons face unique challenges. It remains debated whether damaged axonal mitochondria are transported back to the soma for turnover (Maday *et al*., 2012; Maday & Holzbaur, 2014) or are degraded locally in axons by PINK1‐Parkin‐dependent mitophagy (Ashrafi *et al*., 2014). In a recent study, Sung *et al*. (2016) elegantly demonstrated that the appearance and the regulation of axonal mitochondria and autophagy differ between *in vivo* conditions and *in vitro* setups – autophagosomes and mitochondria did not colocalize in axons of *Drosophila* motor neurons *in vivo*; however, when these neurons were cultured *in vitro*, numerous autophagosomes were colocalized with mitochondria in axons, even in the absence of any drug‐induced mitochondrial damage. In this study, we provide *in vivo* evidence that the PINK1‐Parkin pathway is not required for mitochondrial turnover or axonal integrity in either sensory (wing) or motor (leg) neurons of the adult *Drosophila* during aging. Certainly, we cannot rule out the possibility that PINK1‐Parkin deficiency may still compromise the ultrastructure or function of axonal mitochondria. Interestingly, Devireddy *et al*. (2015) showed that *Pink1* deletion led to a slight decrease in mitochondrial membrane potential and abnormal mitochondrial morphology in the soma of *Drosophila* larval neurons but did not change mitochondrial density or length in axons. Furthermore, the results of climbing and lifespan assays strongly suggest that the PINK1‐Parkin pathway is dispensable for adult neurons at the animal level. And the reduced longevity of the LOF mutants of *Pink1* and *Parkin* (Greene *et al*., 2003; Cha *et al*., 2005; Clark *et al*., 2006; Park *et al*., 2006) is likely due to their essential role in non‐neuronal cells such as muscles. As the difference ‘between neurons and muscles’ may represent a cell type‐specific expression and/or requirement of PINK1‐Parkin, our data do not exclude the possibility that the PINK1‐Parkin pathway plays an essential role in select subtypes of neurons, such as dopaminergic neurons, and thus contributes to the pathogenesis of PD.

The autophagy machinery is required for the final turnover of mitochondria in all mitophagy pathways (Pickrell & Youle, 2015; Wei *et al*., 2015). Our *in vivo* imaging data reveal that, accompanying the accumulation of axonal mitochondria, the number of axonal autophagosomes increases with age. However, mitochondria and autophagosomes are essentially not colocalized in axons *in vivo*, even in aged neurons. This is in sharp contrast to the neuronal cell bodies where colocalization of mitochondria and autophagosomes is frequently observed, lending support to an important role of mitophagy in the soma (Cai *et al*., 2012; Sung *et al*., 2016). Moreover, knockdown of *Atg12* or *Atg17* inhibits axonal autophagy but does not affect mitochondrial turnover or axonal integrity. As all mitophagy pathways rely on the autophagy machinery, our data demonstrate that neither PINK1‐Parkin‐dependent nor PINK1‐Parkin‐independent mitophagy is required for maintaining axonal mitochondria *in vivo*. Although the function of PINK1‐Parkin in axons has been debated (Ashrafi *et al*., 2014; Devireddy *et al*., 2015; Sung *et al*., 2016), our study is the first to show that overall mitophagy is dispensable for axonal maintenance during normal aging (to the best of our knowledge). Nevertheless, blocking autophagy in adult *Drosophila* neurons results in accelerated aging and slightly shortened lifespan, signifying the importance of autophagy in neuronal aging.

Mitochondrial fission–fusion is required for proper axonal projections and synaptic development and is involved in human neurological disorders (Burté *et al*., 2015; Bertholet *et al*., 2016). However, whether fission–fusion constitutes an essential quality control mechanism for mitochondria in axons and how it impacts on neuronal aging compared to mitophagy are unclear. We show that the proper levels of the fission–fusion genes are critically required in axons during aging and that disturbance of the fission–fusion balance in adult neurons dramatically reduces the longevity. When comparing mitophagy and fission–fusion, it is important to bear in mind that axons can be hundreds or even thousands of times longer than the size of the soma. Should neurons routinely turnover axonal mitochondria by mitophagy, the amount of energy it would cost to constantly transport mitochondria from the soma to distal axons and then back to the soma for lysosome‐mediated degradation would be unmanageably enormous and wasteful. Fission–fusion provides an important alternative mechanism to control mitochondrial quality by ‘repairing and reusing’ mitochondria (Schrepfer & Scorrano, 2016). As such, maintaining axonal mitochondria by fission–fusion can be much more ‘cost‐effective’ than mitophagy. This alternative model is especially attractive as damage to axonal mitochondria under physiological conditions during aging is likely mild and gradual.

Together, our *in vivo* data strongly suggest that maintenance of axonal mitochondria does not require mitophagy, whereas mitophagy‐independent mechanisms such as fission–fusion may play a pivotal role in maintaining axonal mitochondria and neural integrity during normal aging. Finally, it is important to point out that there are multiple levels of mitochondrial quality control, including mitochondrial proteases, ubiquitin‐dependent turnover, vesicle transport to lysosomes, and the new emerging mechanisms of mitochondrial ‘expulsion’ that have been recently reported as an alternative means of autophagy and PINK1‐Parkin‐independent turnover (Melentijevic *et al*., 2017). Each of them may be active or inactive under certain conditions, in select types of cells, or at restricted subcellular compartments. Thus, it will be of great interest to investigate the role of mitophagy‐independent mechanisms of mitochondrial quality control in aging in the future.

## Experimental procedures

### Fly stocks

Flies tested in this study were raised on standard cornmeal media and maintained at 25 °C and 60% relative humidity. The following strains were obtained from the Bloomington *Drosophila* Stock Center (BDSC): *w* ^*1118*^ (5905), *Pink1* ^B9^ (34749), *Pink1* ^5^ (51649), *Da*‐Gal4 (5460), *elav‐*GS (43642), UAS‐mCherry‐*Atg8a* (37750), UAS‐*LacZ* (8529), UAS‐*Pink1* (51648), UAS‐*Parkin* (51651), UAS‐RNAi‐*Parkin* (38333), UAS‐RNAi‐*Atg12* (34675), UAS‐RNAi‐*Atg17* (36918), and UAS‐RNAi‐*Marf* (55189). The following strains were obtained from the Tsinghua Fly Center (TFC): UAS‐RNAi‐*Opa1* (THU0811), UAS‐RNAi‐*Drp1* (TH02258.N), *Mef2*‐Gal4 (THJ0244). For neuronal expression (*Dpr*‐Gal4, *D42*‐Gal4, and *elav‐*GS) of long hairpin RNAi lines used in this study, the RNAi‐*luciferase* (31603) was used as a control and a copy of UAS‐*Dcr2* was co‐expressed to boost the knockdown efficiency (Ni *et al*., 2008); for ubiquitous expression (*Da*‐Gal4) or expression in muscles (*Mef2*‐Gal4), no additional UAS‐Dcr2 was needed and the RNAi‐*mCherry* (35785) was used as a control. The UAS‐*Pink1* ^*L464P*^, UAS‐*Parkin* ^*T240R*^ *,* and UAS‐*Parkin* ^*R275W*^ flies are kind gifts from J. Chung, K.L. Lin, and C.H. Chen.

For simultaneous *in vivo* imaging of axonal mitochondria and axon membrane integrity, the following stable fly strain containing multiple transgenes was generated by chromosomal recombination: *yw;dpr‐Gal4,UAS‐mitoGFP/CyO;UAS‐mCD8‐mCherry/TM6B.Tb*.

For simultaneous *in vivo* imaging of axonal mitochondrial and axonal autophagosomes, the following stable fly line containing multiple transgenes was generated by chromosomal recombination: *yw; dpr‐Gal4, UAS‐mCherry‐Atg8a, UAS‐mitoGFP/Cyo*.

### Statistical analysis

Unless otherwise noted, statistical significance in this study is determined by unpaired, two‐tailed Student's *t*‐test with unequal variance at *P <* *0.05, **P <* *0.01, and ***P <* *0.001. Error bars represent standard error of mean (SEM).

Additional experimental procedures are available in Supporting Information.

## Funding

This study is supported by the National Key R&D Program of China (No. 2016YFA0501902), the National Natural Science Foundation of China (No. 31471017 and No. 81671254), and the State High‐Tech Development Plan of China (the ‘863 Program’, No. 2014A020526) to Y.F.

## Author contributions

X.C. planned and performed experiments, analyzed data, and wrote the manuscript. H.W. and Q.W. performed experiments. X.C., H.W., and Z.W. contributed critical reagents. S.Z. and Y.D. provided technical assistance. Y.F. designed and supervised research and wrote the manuscript.

## Conflict of interest

The authors declare no conflict of interest.

## Supporting information


**Fig. S1** Disruption of the PINK1‐Parkin pathway leads to overt abnormal muscle phenotypes.
**Fig. S2** The PINK1‐Parkin pathway is also dispensable in *Drosophila* motor neurons.
**Fig. S3** Upregulation of the PINK1‐Parkin pathway is detrimental to axons *in vivo*.
**Fig. S4** The core ATG gene Atg12 and Atg17 are required for neuronal autophagy.

## References

[acel12654-bib-0001] Ashrafi G , Schlehe JS , LaVoie MJ , Schwarz TL (2014) Mitophagy of damaged mitochondria occurs locally in distal neuronal axons and requires PINK1 and Parkin. J. Cell Biol. 206, 655–670. 25154397 10.1083/jcb.201401070 PMC4151150

[acel12654-bib-0002] Avery MA , Rooney TM , Pandya JD , Wishart TM , Gillingwater TH , Geddes JW , Sullivan PG , Freeman MR (2012) WldS prevents axon degeneration through increased mitochondrial flux and enhanced mitochondrial Ca2+ buffering. Curr. Biol. 22, 596–600. 22425157 10.1016/j.cub.2012.02.043 PMC4175988

[acel12654-bib-0003] Bertholet AM , Delerue T , Millet AM , Moulis MF , David C , Daloyau M , Arnaune‐Pelloquin L , Davezac N , Mils V , Miquel MC , Rojo M , Belenguer P (2016) Mitochondrial fusion/fission dynamics in neurodegeneration and neuronal plasticity. Neurobiol. Dis. 90, 3–19. 26494254 10.1016/j.nbd.2015.10.011

[acel12654-bib-0004] Bratic A , Larsson NG (2013) The role of mitochondria in aging. J. Clin. Invest. 123, 951–957. 23454757 10.1172/JCI64125 PMC3582127

[acel12654-bib-0005] Burté F , Carelli V , Chinnery PF , Yu‐Wai‐Man P (2015) Disturbed mitochondrial dynamics and neurodegenerative disorders. Nat. Rev. Neurol. 11, 11–24. 25486875 10.1038/nrneurol.2014.228

[acel12654-bib-0006] Cai Q , Zakaria HM , Simone A , Sheng ZH (2012) Spatial parkin translocation and degradation of damaged mitochondria via mitophagy in live cortical neurons. Curr. Biol. 22, 545–552. 22342752 10.1016/j.cub.2012.02.005 PMC3313683

[acel12654-bib-0007] Cha GH , Kim S , Park J , Lee E , Kim M , Lee SB , Kim JM , Chung J , Cho KS (2005) Parkin negatively regulates JNK pathway in the dopaminergic neurons of *Drosophila* . Proc. Natl Acad. Sci. USA 102, 10345–10350. 16002472 10.1073/pnas.0500346102 PMC1177361

[acel12654-bib-0008] Chan DC (2012) Fusion and fission: interlinked processes critical for mitochondrial health. Annu. Rev. Genet. 46, 265–287. 22934639 10.1146/annurev-genet-110410-132529

[acel12654-bib-0009] Clark IE , Dodson MW , Jiang C , Cao JH , Huh JR , Seol JH , Yoo SJ , Hay BA , Guo M (2006) *Drosophila* pink1 is required for mitochondrial function and interacts genetically with parkin. Nature 441, 1162–1166. 16672981 10.1038/nature04779

[acel12654-bib-0011] Devireddy S , Liu A , Lampe T , Hollenbeck PJ (2015) The organization of mitochondrial quality control and life cycle in the nervous system *in vivo* in the absence of PINK1. J. Neurosci. 35, 9391–9401. 26109662 10.1523/JNEUROSCI.1198-15.2015 PMC4478254

[acel12654-bib-0012] Dokladny K , Myers OB , Moseley PL (2015) Heat shock response and autophagy–cooperation and control. Autophagy 11, 200–213. 25714619 10.1080/15548627.2015.1009776 PMC4502786

[acel12654-bib-0013] Fang Y , Bonini NM (2015) Hope on the (fruit) fly: the *Drosophila* wing paradigm of axon injury. Neural Regen. Res. 10, 173–175. 25883604 10.4103/1673-5374.152359 PMC4392653

[acel12654-bib-0014] Fang Y , Soares L , Teng X , Geary M , Bonini NM (2012) A novel *Drosophila* model of nerve injury reveals an essential role of Nmnat in maintaining axonal integrity. Curr. Biol. 22, 590–595. 22425156 10.1016/j.cub.2012.01.065 PMC3347919

[acel12654-bib-0015] Fang Y , Soares L , Bonini NM (2013) Design and implementation of *in vivo* imaging of neural injury responses in the adult *Drosophila* wing. Nat. Protoc. 8, 810–819. 23589940 10.1038/nprot.2013.042 PMC4032490

[acel12654-bib-0501] Green DR , Galluzzi L , Kroemer G (2011) Mitochondria and the autophagy‐inflammation‐cell death axis in organismal aging. Science. 333, 1109–1112. 21868666 10.1126/science.1201940 PMC3405151

[acel12654-bib-0016] Greene JC , Whitworth AJ , Kuo I , Andrews LA , Feany MB , Pallanck LJ (2003) Mitochondrial pathology and apoptotic muscle degeneration in *Drosophila* parkin mutants. Proc. Natl Acad. Sci. USA 100, 4078–4083. 12642658 10.1073/pnas.0737556100 PMC153051

[acel12654-bib-0017] Kim NC , Tresse E , Kolaitis RM , Molliex A , Thomas RE , Alami NH , Wang B , Joshi A , Smith RB , Ritson GP , Winborn BJ , Moore J , Lee JY , Yao TP , Pallanck L , Kundu M , Taylor JP (2013) VCP is essential for mitochondrial quality control by PINK1/Parkin and this function is impaired by VCP mutations. Neuron 78, 65–80. 23498974 10.1016/j.neuron.2013.02.029 PMC3683300

[acel12654-bib-0018] Klionsky DJ , *et al* (2016) Guidelines for the use and interpretation of assays for monitoring autophagy (3rd edition). Autophagy 12, 1–222. 26799652 10.1080/15548627.2015.1100356 PMC4835977

[acel12654-bib-0019] Ktistakis NT , Tooze SA (2016) Digesting the expanding mechanisms of autophagy. Trends Cell Biol. 26, 624–635. 27050762 10.1016/j.tcb.2016.03.006

[acel12654-bib-0020] Lee JY , Nagano Y , Taylor JP , Lim KL , Yao TP (2010) Disease‐causing mutations in parkin impair mitochondrial ubiquitination, aggregation, and HDAC6‐dependent mitophagy. J. Cell Biol. 189, 671–679. 20457763 10.1083/jcb.201001039 PMC2872903

[acel12654-bib-0021] López‐Otín C , Blasco MA , Partridge L , Serrano M , Kroemer G (2013) The hallmarks of aging. Cell 153, 1194–1217. 23746838 10.1016/j.cell.2013.05.039 PMC3836174

[acel12654-bib-0022] Maday S , Holzbaur EL (2014) Autophagosome biogenesis in primary neurons follows an ordered and spatially regulated pathway. Dev. Cell 30, 71–85. 25026034 10.1016/j.devcel.2014.06.001 PMC4109719

[acel12654-bib-0023] Maday S , Wallace KE , Holzbaur EL (2012) Autophagosomes initiate distally and mature during transport toward the cell soma in primary neurons. J. Cell Biol. 196, 407–417. 22331844 10.1083/jcb.201106120 PMC3283992

[acel12654-bib-0024] McWilliams TG , Prescott AR , Allen GF , Tamjar J , Munson MJ , Thomson C , Muqit MM , Ganley IG (2016) mito‐QC illuminates mitophagy and mitochondrial architecture *in vivo* . J. Cell Biol. 214, 333–345. 27458135 10.1083/jcb.201603039 PMC4970326

[acel12654-bib-0025] Melentijevic I , Toth ML , Arnold ML , Guasp RJ , Harinath G , Nguyen KC , Taub D , Parker JA , Neri C , Gabel CV , Hall DH , Driscoll M (2017) *C. elegans* neurons jettison protein aggregates and mitochondria under neurotoxic stress. Nature 542, 367–371. 28178240 10.1038/nature21362 PMC5336134

[acel12654-bib-0026] Morsci NS , Hall DH , Driscoll M , Sheng ZH (2016) Age‐related phasic patterns of mitochondrial maintenance in adult *Caenorhabditis elegans* neurons. J. Neurosci. 36, 1373–1385. 26818523 10.1523/JNEUROSCI.2799-15.2016 PMC4728731

[acel12654-bib-0027] Nakamura M , Baldwin D , Hannaford S , Palka J , Montell C (2002) Defective proboscis extension response (DPR), a member of the Ig superfamily required for the gustatory response to salt. J. Neurosci. 22, 3463–3472. 11978823 10.1523/JNEUROSCI.22-09-03463.2002 PMC6758379

[acel12654-bib-0028] Ni J‐Q , Markstein M , Binari R , Pfeiffer B , Liu L‐P , Villalta C , Booker M , Perkins L , Perrimon N (2008) Vector and parameters for targeted transgenic RNA interference in *Drosophila melanogaster* . Nat. Methods 5, 49–51. 18084299 10.1038/nmeth1146 PMC2290002

[acel12654-bib-0029] Osterwalder T , Yoon KS , White BH , Keshishian H (2001) A conditional tissue‐specific transgene expression system using inducible GAL4. Proc. Natl Acad. Sci. USA 98, 12596–12601. 11675495 10.1073/pnas.221303298 PMC60099

[acel12654-bib-0030] Park J , Lee SB , Lee S , Kim Y , Song S , Kim S , Bae E , Kim J , Shong M , Kim JM , Chung J (2006) Mitochondrial dysfunction in *Drosophila* PINK1 mutants is complemented by parkin. Nature 441, 1157–1161. 16672980 10.1038/nature04788

[acel12654-bib-0031] Pich S , Bach D , Briones P , Liesa M , Camps M , Testar X , Palacin M , Zorzano A (2005) The Charcot‐Marie‐Tooth type 2A gene product, Mfn2, up‐regulates fuel oxidation through expression of OXPHOS system. Hum. Mol. Genet. 14, 1405–1415. 15829499 10.1093/hmg/ddi149

[acel12654-bib-0032] Pickrell AM , Youle RJ (2015) The roles of PINK1, parkin, and mitochondrial fidelity in Parkinson's disease. Neuron 85, 257–273. 25611507 10.1016/j.neuron.2014.12.007 PMC4764997

[acel12654-bib-0033] Poole AC , Thomas RE , Yu S , Vincow ES , Pallanck L (2010) The mitochondrial fusion‐promoting factor mitofusin is a substrate of the PINK1/parkin pathway. PLoS ONE 5, e10054. 20383334 10.1371/journal.pone.0010054 PMC2850930

[acel12654-bib-0034] Pryde KR , Taanman JW , Schapira AH (2016) A LON‐ClpP proteolytic axis degrades complex I to extinguish ROS production in depolarized mitochondria. Cell Rep. 17, 2522–2531. 27926857 10.1016/j.celrep.2016.11.027 PMC5177631

[acel12654-bib-0035] Rana A , Rera M , Walker DW (2013) Parkin overexpression during aging reduces proteotoxicity, alters mitochondrial dynamics, and extends lifespan. Proc. Natl Acad. Sci. USA 110, 8638–8643. 23650379 10.1073/pnas.1216197110 PMC3666724

[acel12654-bib-0036] Saxton WM , Hollenbeck PJ (2012) The axonal transport of mitochondria. J. Cell Sci. 125, 2095–2104. 22619228 10.1242/jcs.053850 PMC3656622

[acel12654-bib-0037] Schrepfer E , Scorrano L (2016) Mitofusins, from mitochondria to metabolism. Mol. Cell 61, 683–694. 26942673 10.1016/j.molcel.2016.02.022

[acel12654-bib-0038] Seibler P , Graziotto J , Jeong H , Simunovic F , Klein C , Krainc D (2011) Mitochondrial Parkin recruitment is impaired in neurons derived from mutant PINK1 induced pluripotent stem cells. J. Neurosci. 31, 5970–5976. 21508222 10.1523/JNEUROSCI.4441-10.2011 PMC3091622

[acel12654-bib-0039] Song S , Jang S , Park J , Bang S , Choi S , Kwon KY , Zhuang X , Kim E , Chung J (2013) Characterization of PINK1 (PTEN‐induced putative kinase 1) mutations associated with Parkinson disease in mammalian cells and *Drosophila* . J. Biol. Chem. 288, 5660–5672. 23303188 10.1074/jbc.M112.430801 PMC3581423

[acel12654-bib-0040] Sung H , Tandarich LC , Nguyen K , Hollenbeck PJ (2016) Compartmentalized regulation of parkin‐mediated mitochondrial quality control in the *Drosophila* nervous system *in vivo* . J. Neurosci. 36, 7375–7391. 27413149 10.1523/JNEUROSCI.0633-16.2016 PMC4945662

[acel12654-bib-0041] Takihara Y , Inatani M , Eto K , Inoue T , Kreymerman A , Miyake S , Ueno S , Nagaya M , Nakanishi A , Iwao K , Takamura Y , Sakamoto H , Satoh K , Kondo M , Sakamoto T , Goldberg JL , Nabekura J , Tanihara H (2015) *In vivo* imaging of axonal transport of mitochondria in the diseased and aged mammalian CNS. Proc. Natl Acad. Sci. USA 112, 10515–10520. 26240337 10.1073/pnas.1509879112 PMC4547257

[acel12654-bib-0043] Wai T , Langer T (2016) Mitochondrial dynamics and metabolic regulation. Trends Endocrinol. Metab. 27, 105–117. 26754340 10.1016/j.tem.2015.12.001

[acel12654-bib-0502] Wang K , Klionsky DJ (2014) Mitochondria removal by autophagy. Autophagy. 7, 297–300. 10.4161/auto.7.3.14502 PMC3359476 21252623

[acel12654-bib-0044] Wei H , Liu L , Chen Q (2015) Selective removal of mitochondria via mitophagy: distinct pathways for different mitochondrial stresses. Biochim. Biophys. Acta 1853, 2784–2790. 25840011 10.1016/j.bbamcr.2015.03.013

[acel12654-bib-0045] Ziviani E , Tao RN , Whitworth AJ (2010) *Drosophila* parkin requires PINK1 for mitochondrial translocation and ubiquitinates mitofusin. Proc. Natl Acad. Sci. USA 107, 5018–5023. 20194754 10.1073/pnas.0913485107 PMC2841909

