# Supplementary material for: In vivo imaging reveals mitophagy independence in the maintenance of axonal mitochondria during normal aging
Source: Aging Cell. 2017 Aug 7;16(5):1180–90. doi: 10.1111/acel.12654 (PMC5595681; doi:10.1111/acel.12654)
Supplement: Supplementary file 1 — Fig. S1 Disruption of the PINK1‐Parkin pathway leads to overt abnormal muscle phenotypes. Fig. S2 The PINK1‐Parkin pathway is also dispensable in Drosophila motor neurons. Fig. S3 Upregulation of the PINK1‐Parkin pathway is detrimental to axons in vivo. Fig. S4 The core ATG gene Atg12 and Atg17 are required for neuronal autophagy. [file ACEL-16-1180-s001.pdf]

SUPPLEMENTAL INFORMATION

In vivo imaging reveals mitophagy independence in the maintenance of axonal mitochondria during normal aging

Xu Cao, Haiqiong Wang, Zhao Wang, Qingyao Wang, Shuang Zhang, Yuanping Deng and Yanshan Fang

Figure S1

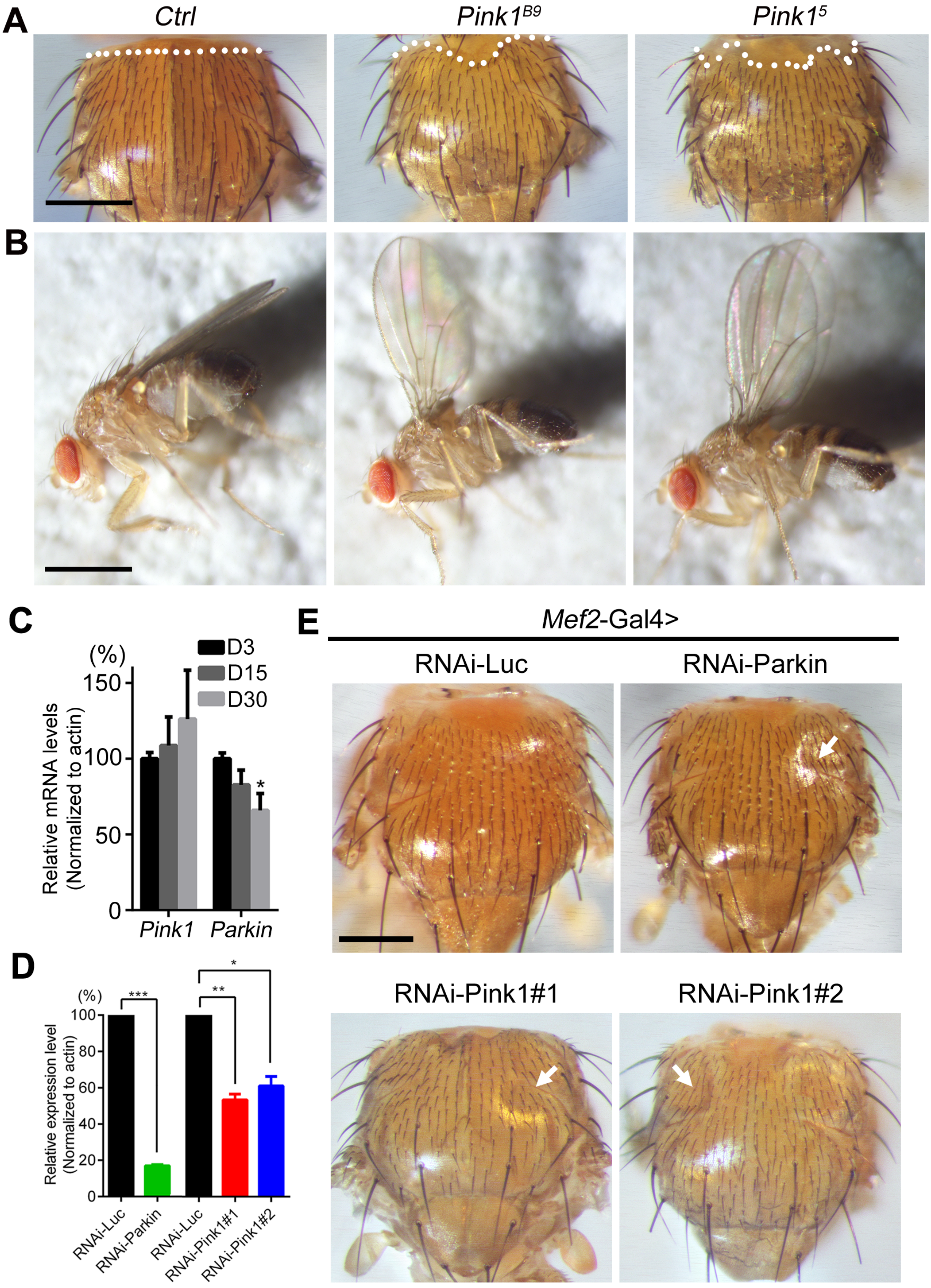

A
Ctrl
Pink1B9
Pink15
B
C
(%)
150
100
50
0
D3
D15
D30
Relative mRNA levels
(Normalized to actin)
*
Pink1
Parkin
D
(%)
100
80
60
40
20
0
***
**
*
Relative expression level
(Normalized to actin)
RNAi-Luc
RNAi-Parkin
RNAi-Luc
RNAi-Pink1#1
RNAi-Pink1#2
E
Mef2-Gal4>
RNAi-Luc
RNAi-Parkin
RNAi-Pink1#1
RNAi-Pink1#2

Figure S1. Disruption of the PINK1-Parkin pathway leads to overt abnormal muscle phenotypes. (A) Representative images of the thorax of the control (w^{1118}), *Pink1*^{B9} and *Pink1*^{5} flies. The *Pink1* mutant flies have an apparent crushed thorax phenotype, as outlined by the white dashed lines. (B) Both *Pink1*^{B9} and *Pink1*^{5} mutant flies show held-up wings compared to control flies. The muscle degenerative phenotypes are consistent with the previous reports of the *Pink1* mutants (Clark, et al., 2006; Park et al., 2006). (C) The relative mRNA levels of *Pink1* and *Parkin* in the fly head at the indicated time points are analyzed by qPCR and normalized to actin. The expression of *Parkin* is significantly reduced at D30. (D) The knockdown efficiency of RNAi-*Parkin* and two independent transgenic lines of RNAi-*Pink1* is determined by qPCR. Data are shown as mean ± SEM. $*p < 0.05$, $**p < 0.01$, $***p < 0.001$. (E) Downregulation of *Parkin* or *Pink1* in muscles (*Mef2*-Gal4) using the same RNAi transgenic strains of the neuronal manipulations (see Fig. 2E-2H) causes muscle degenerative phenotypes including collapsed thorax (arrow), which is consistent with previous reports of the LOF mutants of *Parkin* (Greene et al., 2003; Cha et al., 2005) and *Pink1* (Clark, et al., 2006; Park et al., 2006). Scale bars, 500 µm in (A and E) and 1 mm in (B).

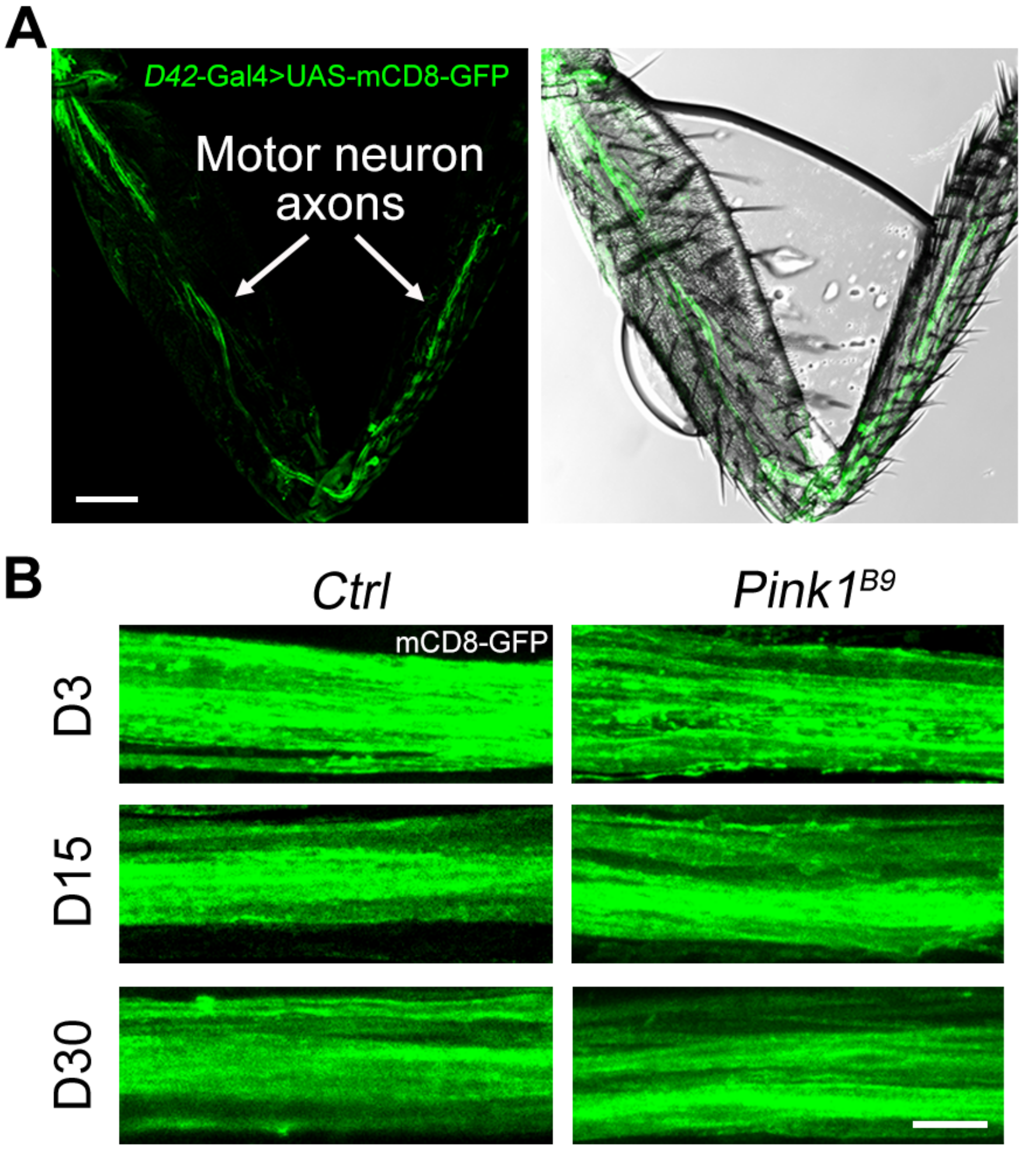

Figure S2. The PINK1-Parkin pathway is also dispensable in *Drosophila* motor neurons

(A) Representative images of motor neuron axons labeled by mCD8-GFP (left) using a *D42*-Gal4 driver in the fly leg (right, merged with bright field). (B) Motor neuron axons of control (w^{1118}) and $Pink1^{B9}$ at D3, D15 and D30. Similar to sensory neurons in the wing nerve (Fig. 2D), no axonal degeneration is observed in the LOF mutants of *Pink1*. Scale bars, 50 µm in (A) and 5 µm in (B).

Figure S3

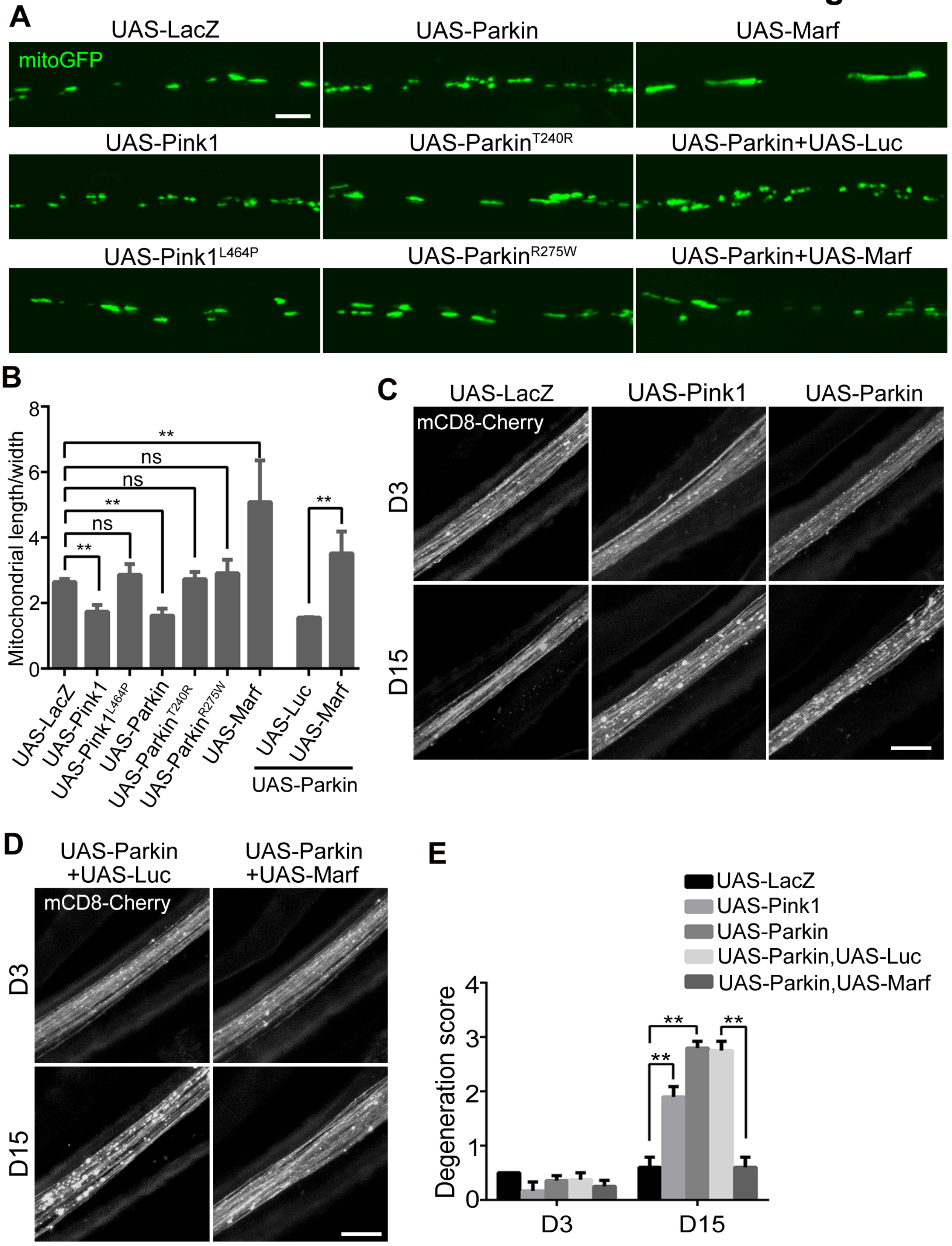
A
UAS-LacZ
UAS-Parkin
UAS-Marf
mitoGFP
UAS-Pink1
UAS-ParkinT240R
UAS-Parkin+UAS-Luc
UAS-Pink1L464P
UAS-ParkinR275W
UAS-Parkin+UAS-Marf
B
Mitochondrial length/width
ns
**
UAS-LacZ
UAS-Pink1
UAS-Pink1L464P
UAS-Parkin
UAS-ParkinT240R
UAS-ParkinR275W
UAS-Marf
UAS-Luc
UAS-Marf
UAS-Parkin
C
UAS-LacZ
UAS-Pink1
UAS-Parkin
mCD8-Cherry
D3
D15
D
UAS-Parkin +UAS-Luc
UAS-Parkin +UAS-Marf
mCD8-Cherry
D3
D15
E
UAS-LacZ
UAS-Pink1
UAS-Parkin
UAS-Parkin,UAS-Luc
UAS-Parkin,UAS-Marf
Degeneration score
D3
D15

Figure S3. Upregulation of the PINK1-Parkin pathway is detrimental to axons *in vivo*.

(A) Axonal mitochondria in the costal wing nerve of the indicated genotypes. The mitochondrial length/width ratio is quantified in (B). Data are shown as mean ± SEM, n = 7~10 wings per group. ***$p < 0.001$. (C-D) Representative *in vivo* images of mCD8-mCherry labeled L1 wing nerve of indicated genotypes at D3 and D15. (E) Quantification of the axonal degeneration scores of (C) and (D) as described in the Methods. Data are shown as mean ± SEM, n = 6~8 wings per group. Scale bars, 5 μm in (A) and 10 μm in (C-D). Overexpression of *Pink1* or *Parkin* in the wing axons led to shortened mitochondria, while upregulation of the mitochondrial fusion gene *Marf* increased mitochondrial length. The transgenic expression of catalytically inactive *Pink1* mutants, *Pink1*^{L464P} (Song et al., 2013), and *Parkin* mutants, *Parkin*^{T240R} or *Parkin*^{R275W} (Lee et al., 2010; Kim et al., 2013), using the same Gal4 driver did not significantly alter the mitochondrial morphology in axons, suggesting that the mitochondrial fragmentation and detrimental effects of overexpressing *Pink1* or *Parkin* require their enzymatic activity in this context. In addition, both mitochondrial morphology and axonal integrity of the *Parkin*-overexpressing flies are improved by co-expression of the mitochondrial fusion gene *Marf*.

Figure S4

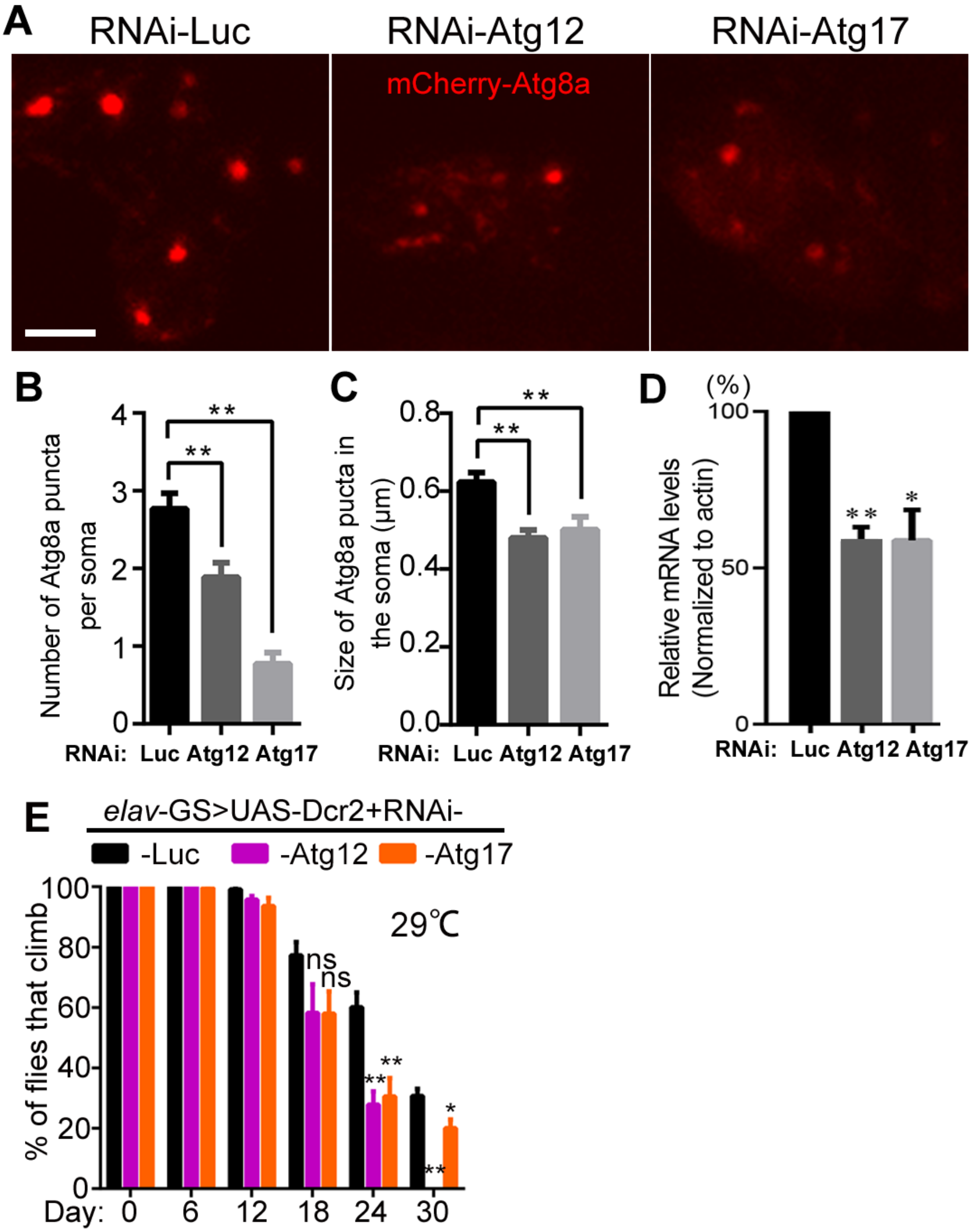
A
RNAi-Luc
RNAi-Atg12
RNAi-Atg17
mCherry-Atg8a
B
Number of Atg8a puncta per soma
RNAi: Luc Atg12 Atg17
**
C
Size of Atg8a pucta in the soma (μm)
RNAi: Luc Atg12 Atg17
D
(%)
Relative mRNA levels (Normalized to actin)
RNAi: Luc Atg12 Atg17
E
elav-GS>UAS-Dcr2+RNAi-
-Luc
-Atg12
-Atg17
29℃
% of flies that climb
ns
Day: 0 6 12 18 24 30

Figure S4. The core ATG genes *Atg12* and *Atg17* are required for neuronal autophagy.

(A) The neuronal soma of the wing nerve show autophagosomes labeled by mCherry-Atg8a of indicated genotypes at D3. Scale bar, 2.5 μm. (B-C) The number and the size of mCherry-Atg8a puncta in the neuronal soma are determined using the "analyze particle" tool of ImageJ and quantified in (B) and (C), respectively. Data are shown as mean ± SEM, n = 11~14 wings per group. (D) The knockdown efficiency of RNAi-*Atg12* and RNAi-*Atg17* is determined by qPCR. n = 20 flies per vial and 6-7 vials group. (E) Climbing assays of flies of indicated genotypes aged at 29 °C. The locomotive ability is assessed as the average percentage of flies climbing over a distance of 5 cm within 15 seconds. Data are shown as mean ± SEM, n = 20 flies per vial and 6-8 vials each group. *$p < 0.05$ **$p < 0.01$. ns, not significant. Compared to the RNAi-*luc* controls, the RNAi-*Atg12* and RNA-*Atg17* flies under heat stress showed dramatically accelerated decline of climbing ability at ages D24 and D30.

SUPPLEMENTAL EXPERIMENTAL PROCEDURES

Generation of Transgenic *Drosophila* Strains

Six tandem *c-myc* epitopes were amplified by routine PCR and subcloned into the pBID-UASC vector (Wang et al., 2012) between the KpnI and XbaI sites using ClonExpress^{TM} II One Step Cloning Kit (Vazyme). The coding sequence of *Drosophila Marf* (RE04414) was retrieved from cDNA clones from the Drosophila Genomics Resource Center (DGRC) and sub-cloned into pBID-UASC-6*myc between the EcoRI and XhoI sites. The transgenic fly strain of pBID-UAS-*Marf* (together with pBID-UASC-*Luciferase* as a control) was generated by ΦC31 integrase-mediated, site-specific integration into the fly genome, which allowed uniform transgene expression across different lines. The attP landing site stock used in this study was UAS-phi2b2a; VK5 (75B1).

Wing Nerve Imaging Procedure

The *Drosophila* wing nerve labeled by various fluorescent protein markers was visualized on live flies under a Leica DM6000B fluorescent microscope as described previously (Fang et al., 2013). In order to use a Leica TCS SP8 confocal microscope to obtain images of better quality for publication, wings were detached from the fly body and briefly washed with PBST (0.2% Triton-X-100, to eliminate hydrophobicity of the wing cuticle). Rinsed wings were mounted directly in Vectashield Mounting Medium (Vector Laboratories) and imaged immediately. Similar imaging settings were applied for comparison across different groups.

Imaging Acquisition, Processing and Analysis

Neuronal mitochondria and autophagosomes were labeled by mitoGFP and mCherry-Atg8a, respectively. Images were taken with a confocal microscope (Leica TCS SP8) using a 63X oil objective (NA=1.4). The images were processed and analyzed in LAS X and ImageJ, and assembled into figures using Adobe Photoshop CS6 with similar settings. The outline of the Costal or the L1 nerve in each image was drawn as a region of interest (ROI). The signal of mitochondria or autophagosomes in the ROI was defined and the threshold was adjusted to make the machine-identified mitochondria or autophagosome match the

actual morphology in the image. The size and number of mitochondria or autophagosomes were measured using the "Analyze Particles" feature of ImageJ, and their colocalization was determined using the "Colocalization Highlighter" plugin of ImageJ. The axonal integrity was imaged using mCD8-mCherry as a marker and quantified as previously described (Fang et al., 2012). Briefly, in intact axons, the mCD8-Cherry signal appeared mostly smooth and continuous (degeneration score 0). With degeneration, the axonal mCD8-Cherry became blobby (degeneration score 1), fragmented (degeneration score 2), completely broken (degeneration score 3), and eventually lost fluorescent signal (degeneration score 4). The numbers of wings quantified in each experiment were specified in the figure legends.

Quantitative Reverse Transcription (RT)-PCR (qPCR)

The knockdown efficiency of the RNAi lines was determined by extracting total RNA from the whole fly or the fly head of 7-day old adult flies, followed by qPCR assays. A pan-neuronal *elav*-Gal4 driver was used to drive the expression of the UAS-RNAi transgene in all neurons including the wing nerve. For lines that pan-neuronal expression caused early lethality, an eye-specific driver GMR-Gal4 was used and the RNA was extracted from the fly heads only. UAS-RNAi-luc was used as a control with the *elav*-Gal4 or GMR-Gal4 driver, respectively. Approximately 30 flies were collected for each test and 3 biological repeats were performed for the statistical analysis. For qPCR, total RNA was isolated from whole flies or fly heads using TRIzol (Invitrogen) according to the manufacturer's instruction. After DNase (Promega) treatment to remove genomic DNA, RT reactions were performed using High-Capacity cDNA Reverse Transcription kit (Applied Biosystems) with random primers. The cDNA was then used for real-time PCR with SYBR Select Master Mix (Applied Biosystems) by QuantStudio™ 6 Flex Real-Time PCR system (Life Technologies). *actin* was used as an internal reference to normalize the mRNA levels of genes of interest. The following qPCR primers were used:

actin forward: 5'---GAGCGCGGTTACTCTTTCAC---3'

actin reverse: 5'--- GCCATCTCCTGCTCAAAGTC ---3'

Pink1 forward: 5'--- CAGGAACAAGAGCAGCATCA ---3'

Pink1 reverse: 5'--- AGAGCGTTGCTCTGGATGTC ---3'

Parkin forward: 5'--- TTGTACGCAAAATGCTGGAG ---3'

Parkin reverse: 5'--- GTGCCACCAGTTCCTTTACG ---3'

Atg12 forward: 5'---CAGCGAGCAAATTTTCCTGT---3'

Atg12 reverse: 5'--- CACGCCTGATTCTTGCAGTA---3'

Atg17 forward: 5'--- GAGCTCAGCCAGGAGAAGAG---3'

Atg17 reverse: 5'--- CATCCTTCCAAGGCGATAGA---3'

Marf forward: 5'--- GTCCATGAGACGACCACCTT---3'

Marf reverse: 5'--- GAAGGCCACCTTCATGTGAT---3'

Opa1 forward: 5'--- TGGTAAAACCGTCAGCAATG ---3'

Opa1 reverse: 5'--- TTGGTCATCTGGTGAATGGA ---3'

Drp1 forward: 5'--- ACCCTGGACTCCATTCACC ---3'

Drp1 reverse: 5'--- GAATCTGGCGCTTCACTAGC ---3'

Lifespan and Climbing Assays

For lifespan experiments, 20 flies per vial, 8-11 vials per group were tested. Flies were transferred to fresh fly food every 3 days. Flies lost prior to natural death through escape or accidental death were excluded from the final analysis. The median lifespan was calculated as the mean of the medians of each vial of the same group, whereas the "50% survival" shown on the survival curves was derived from compilation of all vials of the group. Thus, there was sometimes a slight difference from these two calculations but the conclusions were not affected. For the climbing assays, 20 flies were transferred into an empty polystyrene vial and gently tapped down to the bottom of the vial. The number of flies that climbed over a distance of 5 cm within 15 seconds was recorded. The test was repeated three times for each vial and 6~8 vials per group were tested. For adult-onset, neuronal expression of the RNAi transgenes using the *elav*-GS driver (Osterwalder et al., 2001), flies were raised at 25 °C and 60% relative humidity on regular fly food supplemented with 80 μg/ml RU486 (TCI).

SUPPLEMENTAL REFERENCES

Cha, G.H., Kim, S., Park, J., Lee, E., Kim, M., Lee, S.B., Kim, J.M., Chung, J., and Cho, K.S. (2005). Parkin negatively regulates JNK pathway in the dopaminergic neurons of *Drosophila*. Proc. Natl Acad. Sci. USA *102*, 10345-10350.

Clark, I.E., Dodson, M.W., Jiang, C., Cao, J.H., Huh, J.R., Seol, J.H., Yoo, S.J., Hay, B.A., and Guo, M. (2006). Drosophila pink1 is required for mitochondrial function and interacts genetically with parkin. Nature *441*, 1162-1166.

Fang Y, Soares L, Teng X, Geary M, Bonini NM (2012) A novel Drosophila model of nerve injury reveals an essential role of Nmnat in maintaining axonal integrity. Curr. Biol. *22*, 590-595.

Fang Y, Soares L, Bonini NM (2013) Design and implementation of in vivo imaging of neural injury responses in the adult Drosophila wing. Nat. Protoc. *8*, 810-819.

Greene, J.C., Whitworth, A.J., Kuo, I., Andrews, L.A., Feany, M.B., and Pallanck, L.J. (2003). Mitochondrial pathology and apoptotic muscle degeneration in *Drosophila* parkin mutants. Proc. Natl Acad. Sci. USA *100*, 4078-4083.

Kim, N.C., Tresse, E., Kolaitis, R.M., Molliex, A., Thomas, R.E., Alami, N.H., Wang, B., Joshi, A., Smith, R.B., Ritson, G.P., et al. (2013). VCP is essential for mitochondrial quality control by PINK1/Parkin and this function is impaired by VCP mutations. Neuron 78, 65-80.

Lee, J.Y., Nagano, Y., Taylor, J.P., Lim, K.L., and Yao, T.P. (2010). Disease-causing mutations in parkin impair mitochondrial ubiquitination, aggregation, and HDAC6-dependent mitophagy. J. Cell Biol. 189, 671-679.

Osterwalder T, Yoon KS, White BH, Keshishian H (2001) A conditional tissue-specific transgene expression system using inducible GAL4. Proc. Natl Acad. Sci. USA *98*, 12596-12601.

Park, J., Lee, S.B., Lee, S., Kim, Y., Song, S., Kim, S., Bae, E., Kim, J., Shong, M., Kim, J.M., *et al.* (2006). Mitochondrial dysfunction in *Drosophila* PINK1 mutants is complemented by parkin. Nature *441*, 1157-1161.

Song, S., Jang, S., Park, J., Bang, S., Choi, S., Kwon, K.Y., Zhuang, X., Kim, E., and Chung, J. (2013). Characterization of PINK1 (PTEN-induced putative kinase 1) mutations associated with Parkinson

disease in mammalian cells and *Drosophila*. J. Biol. Chem. *288*, 5660-5672.

Wang JW, Beck ES, McCabe, B.D (2012) A modular toolset for recombination transgenesis and neurogenetic analysis of *Drosophila*. PloS ONE *7*:e42102.
